# Genomic surveillance uncovers high-risk resistance profiles in *Escherichia coli* and *Klebsiella* spp. from Central Mexico

**DOI:** 10.3389/fcimb.2026.1939440

**Published:** 2026-10-21

**Authors:** Miriam Sarahi Lozano Gamboa, Brian Muñoz Gomez, Andrea Yaxiri Maldonado Gonzaga, Moisés Alejandro Alejo Hernández, Adrian Cazares, Daniel Diaz, Anastacio Palacios Marmolejo, Corina Diana Ceapă

**Affiliations:** 1 Laboratorio Estatal de Salud Pública del Estado de Aguascalientes (LESPA), Aguascalientes, Mexico; 2 Microbiology Laboratory, Department of Natural Products Chemistry, Institute of Chemistry, National Autonomous University of Mexico (UNAM), CDMX, Mexico; 3 Parasites and Microbes Programme, Wellcome Sanger Institute, Hinxton, United Kingdom; 4 European Molecular Biology Laboratory European Bioinformatics Institute (EMBL-EBI), Wellcome Genome Campus, Hinxton, United Kingdom; 5 Public Health Intelligence Unit, National Institute of Public Health, Cuernavaca, Morelos, Mexico; 6 School of Medicine, Cuauhtemoc University, Aguascalientes Campus, Aguascalientes, Mexico

**Keywords:** antimicrobial resistance, genomic epidemiology, Mexico, mobilome, stewardship, surveillance

## Abstract

**Objective:**

To generate actionable genomic evidence for antimicrobial-resistance (AMR) surveillance in Central Mexico by characterizing the diversity, resistome, and mobilome of multidrug-resistant (MDR) and extensively drug-resistant (XDR) *Escherichia coli, Klebsiella pneumoniae*, and *K. michiganensis* belonging to the *K. oxytoca complex (KoxSC)* circulating between 2021 and 2023, and by contextualizing these findings within national genomic data.

**Design/Methods:**

We sequenced 65 MDR/XDR isolates referred to the State Public Health Laboratory of Aguascalientes (LESPA) and compared them with 268 publicly available Mexican genomes. Phenotypic susceptibility profiles (BD Phoenix M50) were integrated with genomic predictions to assess concordance. Phylogenomics, pangenomics, resistome and mobilome profiling, and virulence analyses were performed using standardized pipelines. Because LESPA receives referral cases from severe or treatment-refractory infections, this dataset represents a high-risk clinical subset rather than a population-level sample.

**Results:**

The sequenced isolates displayed substantial lineage diversity (34 sequence types across *E. coli* and *Klebsiella* spp.) and more than 100 distinct antimicrobial-resistance gene combinations. Several determinants not previously reported in Mexican resistant microorganisms—such as *TEM-116*, *aadA22*, *ermB*, and *uhpA*—were identified. Carbapenemase NDM-1 and colistin-associated MCR-9.1 were widespread, and resistance genes frequently co-localized with mobile genetic elements, suggesting active horizontal gene-transfer dynamics. Post-2020 isolates exhibited increased resistome richness, higher XDR rates, and more frequent coinfections, particularly in critical-care settings. The *K. michiganensis* isolates represent the first MDR genomes of this species from Aguascalientes and contribute to the expanding genomic characterization of this underrepresented member of the *K. michiganensis* (KoxSC) in Mexico.

**Conclusions:**

This study provides the first genomic baseline for MDR *E. coli* and *Klebsiella* spp. in Central Mexico and documents an increasingly complex resistome likely shaped by clinical practices, referral patterns, and structural limitations in microbiological capacity. Embedding real-time whole-genome sequencing into routine diagnostics and public-health workflows is essential to strengthen stewardship, guide infection control, and support equitable AMR surveillance across Mexico.

## Introduction

The global rise of antimicrobial-resistant *E. coli* and *Klebsiella* spp. continues to challenge clinical management and public-health systems, particularly in regions where microbiological infrastructure and genomic surveillance remain limited.

*Escherichia coli* and *Klebsiella* species are among the most frequent causes of urinary tract infections, pneumonia, sepsis, and neonatal infections worldwide, and their increasing resistance to first-line and last-resort antibiotics has been linked to the dissemination of extended-spectrum β-lactamases and carbapenemase-encoding genes.

Although large-scale genomic initiatives have mapped the spread of high-risk lineages in Europe, Asia, and North America, surveillance coverage remains uneven, with Latin America consistently underrepresented in global datasets (Wyres et al., 2020; Murray et al., 2022; Naghavi et al., 2024).

Recent worldwide genomic surveillance has revealed the rise of carbapenem-resistant *K. pneumoniae* and ESBL-producing *E. coli*, frequently associated with plasmid-mediated dissemination of *blaKPC, blaNDM*, and *blaCTX-M* variants (Lam et al., 2021; Yaikhan et al., 2024). Genomic studies from Mexico and other Latin American settings have begun to document the emergence of these microorganisms in hospital settings (Magaña-Lizárraga et al., 2019; Merida-Vieyra et al., 2019; Faccone et al., 2023; Chicoski et al., 2025). Complementary systematic reviews adopting a One Health perspective show substantial ESBL burdens in *E. coli* and *K. pneumoniae* across human, animal, and environmental reservoirs, underscoring the need for integrated surveillance frameworks.

Despite these regional advances, genomic surveillance in Latin America remains fragmented, often limited to outbreak investigations or single-center collections, and rarely includes longitudinal sampling. In contrast to emerging datasets from other countries—such as comprehensive ESBL *E. coli* studies in Italy and Spain—publicly available genomic data for *E. coli* and *Klebsiella* spp.in Mexico are sparse, geographically concentrated, and lack representation from several central states. Genomic data for *K. michiganensis* in Mexico remain scarce, with only one clinical isolate previously reported (Alvarado-Delgado et al., 2023).

These gaps are particularly relevant in the context of the COVID-19 pandemic, which reshaped hospital dynamics, antibiotic-use practices, and referral patterns across the country.

State-level public-health laboratories, including the State Public Health Laboratory of Aguascalientes (LESPA), receive isolates primarily from severe or treatment-refractory infections, creating an inherent referral bias toward highly resistant strains, a pattern increasingly recognized in hospital-based genomic surveillance studies (Magaña-Lizárraga et al., 2019; Faccone et al., 2023; Chicoski et al., 2025). Recognizing this bias is essential for interpreting genomic data generated from such collections, which represent a high-risk clinical subset rather than a population-level sample.

In this study, we address critical surveillance gaps by generating and analyzing whole-genome sequences of multidrug-resistant *E. coli, K. pneumoniae*, and *K. michiganensis* (KoxSC) collected in Central Mexico between 2021 and 2023. By integrating phenotypic susceptibility testing with phylogenomics, resistome and mobilome profiling, and comparison against all publicly available Mexican genomes, we characterize the diversity, gene-transfer dynamics, and emerging resistance patterns circulating in the region. Our findings provide the first genomic baseline for *E. coli* and *Klebsiella* spp. in Aguascalientes and reveal increasing resistome complexity in post-2020 isolates. Beyond their scientific value, these results underscore the urgent need to embed real-time genomic surveillance into routine diagnostics and public-health decision-making to strengthen stewardship, guide infection control, and support equitable AMR surveillance across Mexico.

## Methods

### Ethics and biosafety

This study was conducted using clinical isolates collected as part of routine diagnostic procedures in public hospitals in the state of Aguascalientes, Mexico. Ethical approval for the genomic analysis of *Escherichia coli, Klebsiella pneumoniae*, and *Klebsiella michiganensis (*KoxSC) isolates collected between March 2021 and December 2023 was obtained from the Promotora Médica Aguascalientes S.A. de C.V. Ethics and Biosafety Committees (Approval Nos. 100.cbpma.2025 and 2937.ceipma.2025), registered with COFEPRIS. Informed consent was obtained according to Mexican regulations, and annual progress reports were submitted to ensure ongoing compliance with biosafety and clinical research standards. All procedures adhered to WHO guidelines for antimicrobial resistance surveillance and international ethical frameworks for genomic data sharing (PAHO/WHO, 2023).

### Clinical samples

Clinical specimens were obtained from hospitalized patients with suspected or confirmed multidrug-resistant infections, reflecting a referral-based model that enriches the dataset for severe or treatment-refractory cases. Upon receipt at the State Public Health Laboratory of Aguascalientes (LESPA), isolates were cultured under standard conditions and identified to species level using MALDI-TOF MS (MALDI Biotyper^®^, Bruker Daltonics). Antimicrobial susceptibility testing was performed using the BD Phoenix™ M50 system. Susceptibility was assessed for 23 antimicrobial agents across 12 classes: aminoglycosides (amikacin, gentamicin), β-lactams (ampicillin, amoxicillin-clavulanate, ampicillin-sulbactam, piperacillin-tazobactam), cephalosporins (cefazolin, cefuroxime, cefoxitin, ceftriaxone, cefepime), carbapenems (imipenem, meropenem, ertapenem), fluoroquinolones (ciprofloxacin, levofloxacin), monobactams (aztreonam), nitrofurans (nitrofurantoin), glycylcyclines (tigecycline), sulfonamides (trimethoprim-sulfamethoxazole), macrolides (azithromycin), phenicols (chloramphenicol), and tetracyclines (tetracycline). Susceptibility results were interpreted according to CLSI Performance Standards for Antimicrobial Susceptibility Testing, 31st edition (CLSI M100-ED31; 2021).

### Strain selection for whole-genome sequencing

Between 2021 and 2023, LESPA processed 3,454 *E. coli* and *Klebsiella* spp. isolates, including 683 *E. coli*, 679 *K. pneumoniae*, and 92 isolates identified as *K. oxytoca* by MALDI-TOF and BD Phoenix™ M50, of which 2 were subsequently reclassified as *K. michiganensis* through whole-genome sequencing. Although LESPA processed isolates from multiple Enterobacterales genera during the study period, *E. coli*, *K. pneumoniae* and K. michiganensis were the predominant MDR/XDR species identified and were therefore prioritized for genomic characterization. Emergent clinical MDR species with much fewer isolations and antimicrobial resistant genes (ARG), such as *K. aerogenes*, will be described separatelly. Of these, 246 met multidrug-resistant (MDR) or extensively drug-resistant (XDR) criteria, defined as non-susceptibility to at least one agent in three or more antimicrobial classes for MDR, and non-susceptibility to all but two or fewer classes for XDR. From this pool, 65 isolates were selected for whole-genome sequencing (**Supplementary Table S1**). Selection was based on confirmed species identification, availability of a pure and clinically relevant culture, and fulfillment of DNA quality requirements, including an A260/A280 ratio of 1.8–2.0 and a minimum concentration of 100 ng/µL as measured by BioDrop Duo+ and Qubit™ dsDNA HS Assay. This selection reflects both biological suitability and the inherent referral-based enrichment for highly resistant isolates. Clinical metadata for all sequenced isolates are provided in **Supplementary Table S1.**

### DNA extraction and genomic sequencing

Genomic DNA was extracted from single-colony cultures using the QIAsymphony Mericon Bacteria Kit (QIAGEN). DNA purity and concentration were assessed using BioDrop Duo+ and Qubit™ dsDNA HS Assay. Sequencing libraries were prepared with the Illumina DNA Prep kit and sequenced on an Illumina MiniSeq platform using paired-end 2×150 bp chemistry. Raw reads were quality-filtered and trimmed with fastp, and assemblies were generated using SPAdes v3.15.1 (Bankevich et al., 2012) with the “--careful” option. Assembly quality was evaluated using CheckM, and metrics including genome size, GC content, contig number, N50/L50, and completeness/contamination are reported in **Supplementary Table S2**. Genome assemblies were required to meet minimum thresholds of ≥30× mean coverage, and ≤5% contamination, with the exception of two genomes for strains, KpLESPA_199 and KpLESPA_208, that have contamination levels above 10%, which we considered valuable enough to be included.

### Selection of public genomes

To contextualize the LESPA isolates within the broader Mexican genomic landscape, publicly available *E. coli* and *Klebsiella* spp. genomes were retrieved from the BV-BRC v3.30.19 (Olson et al., 2023) platform (October 2024). BV-BRC was selected due to its standardized re-annotation pipeline and curated metadata for pathogen genomes. Genomes were included if they originated from human hosts in Mexico, were classified as complete or whole-genome shotgun assemblies, and met the predefined quality thresholds, including fewer than 200 contigs, GC content between 48% to 57% for *E. coli*, 55% to 59% for *Klebsiella* sp., an estimated contamination level below 10%, and a total genome size between 4 and 6 Mb. Because only limited *K. michiganensis* genomes from Mexico were available at the time of the original analysis, worldwide high-quality *K. michiganensis* genomes passing our quality filters were retrieved from BV-BRC and NCBI. This yielded 41 global *K. michiganensis* genomes, which together with the 5 Mexican isolates (2 LESPA and 3 publicly available) constitute the 46-genome comparative dataset. Metadata for all 268 public genomes are provided in **Supplementary Table S3**.

### Bioinformatics analyses

All 333 genomes (65 LESPA and 268 public) were re-annotated using Prokka v1.14.6 (Seemann, 2014). Pangenome analysis was performed using Roary v3.13.0 (Page et al., 2015) with a BLASTP identity threshold of 95%. Core genes were defined as those present in ≥99% of genomes per species, soft core as 95–99%, shell as 15–95%, and cloud as <15%. Core-gene alignments were used to infer maximum-likelihood phylogenies with IQ-TREE v2.4.0 (Minh et al., 2020). ModelFinder was used to select the best-fit substitution model according to the Bayesian Information Criterion, and branch support was assessed with 1,000 ultrafast bootstrap replicates. Phylogenies were visualized and annotated in iTOL v6.

Sequence types were assigned using MLST v2.22.0 (Galaxy platform, PubMLST database). Taxonomic identity of the two LESPA KoxSC isolates was confirmed using the Type Strain Genome Server (TYGS) (Meier-Kolthoff and Göker, 2019), incorporating all available high-quality *K. michiganensis* reference genomes worldwide. Antimicrobial resistance genes were identified using BLAST searches against CARD (Alcock et al., 2020) (2024 release), NDARO, and SraX, applying thresholds of ≥90% identity and ≥80% coverage. A consolidated resistome matrix for all genomes is provided in **Supplementary Tables S4**-**S6**.

Genotype–phenotype concordance was assessed by comparing BD Phoenix™ M50 susceptibility results with genomic predictions. Concordance was defined as phenotypic resistance accompanied by the corresponding resistance gene presence in each genome, or phenotypic susceptibility in the absence of such genes. Intermediate phenotypes were excluded, and intrinsic resistance determinants were not considered discordant. We acknowledge that concordance may be influenced by factors beyond gene presence, including gene expression levels, regulatory mutations, epistatic interactions, promoter efficiency, and experimental testing conditions.

Mobile genetic elements were identified using MGE-Finder with default parameters. A total of 211 MGEs (mobile genetic element) in *Klebsiella* *pneumoniae* and 345 in *E. coli* LESPA isolates were catalogued (**Supplementary Table S7**). Associations between MGEs and resistance genesi were visualized using SankeyMATIC. Virulence genes were identified using BLAST searches against the VICTORS, with thresholds of ≥90% identity and ≥80% coverage, and profiles for *E. coli* and *K. pneumoniae* are provided in **Supplementary Table S8**.

### Data and strain availability

All genomes generated in this study have been deposited in the NCBI database under BioProject PRJNA1051187 (**Supplementary Tables S1**, **S2**). Detailed protocols and command-line parameters are provided in the **Supplementary Methodology**. Physical isolates are preserved in the Microbiology Laboratory Pathogen Strain Collection at the Institute of Chemistry, UNAM, and are available upon request. Individual BioSample accession numbers are as follows: KpLESPA_30 (SAMN38876756), KpLESPA_89 (SAMN38876757), KpLESPA_92 (SAMN38876758), KpLESPA_95 (SAMN38876759), KpLESPA_99 (SAMN38876760), KpLESPA_108 (SAMN38876761), KpLESPA_110 (SAMN38876762), KpLESPA_2 (SAMN38876763), KpLESPA_3 (SAMN38876764), KpLESPA_139 (SAMN38876765), KpLESPA_144 (SAMN38876766), KpLESPA_146 (SAMN38876767), KpLESPA_148 (SAMN38876768), KpLESPA_153 (SAMN38876769), KpLESPA_178 (SAMN38876770), KoLESPA_188 (SAMN38876771), KpLESPA_190 (SAMN38876772), KpLESPA_193 (SAMN38876773), KpLESPA_196 (SAMN38876774), KpLESPA_197 (SAMN38876775), KpLESPA_199 (SAMN38876776), KoLESPA_200 (SAMN38876777), KpLESPA_203 (SAMN38876778), KpLESPA_204 (SAMN38876779), KpLESPA_208 (SAMN38876780), KpLESPA_209 (SAMN38876781), KpLESPA_210 (SAMN38876782), KpLESPA_211 (SAMN38876783), KpLESPA_214 (SAMN38876784), KpLESPA_215 (SAMN38876785), KpLESPA_216 (SAMN38876786), KpLESPA_218 (SAMN38876787), KpLESPA_219 (SAMN38876788), KpLESPA_220 (SAMN38876789), KpLESPA_240 (SAMN38876790), EcLESPA_17 (SAMN38876791), EcLESPA_71 (SAMN38876792), EcLESPA_72 (SAMN38876793), EcLESPA_79 (SAMN38876794), EcLESPA_82 (SAMN38876795), EcLESPA_83 (SAMN38876796), EcLESPA_96 (SAMN38876797), EcLESPA_107 (SAMN38876798), EcLESPA_112 (SAMN38876799), EcLESPA_114 (SAMN38876800), EcLESPA_118 (SAMN38876801), EcLESPA_119 (SAMN38876802), EcLESPA_121 (SAMN38876803), EcLESPA_122 (SAMN38876804), EcLESPA_127 (SAMN38876805), EcLESPA_130 (SAMN38876806), EcLESPA_135 (SAMN38876807), EcLESPA_136 (SAMN38876808), EcLESPA_160 (SAMN38876809), EcLESPA_166 (SAMN38876810), EcLESPA_169 (SAMN38876811), EcLESPA_183 (SAMN38876812), EcLESPA_184 (SAMN38876813), EcLESPA_187 (SAMN38876814), EcLESPA_192 (SAMN38876815), EcLESPA_195 (SAMN38876816), EcLESPA_205 (SAMN38876817), EcLESPA_207 (SAMN38876818), EcLESPA_238 (SAMN38876819), EcLESPA_239 (SAMN38876820).

## Results

### Clinical and epidemiological features of the LESPA isolates

Between 2021 and 2023, the State Public Health Laboratory of Aguascalientes received 3,454 *E. coli* and *Klebsiella* spp. isolates from regional hospitals. Among these, 246 met MDR or XDR criteria, and 65 were selected for whole-genome sequencing. The sequenced collection comprised 30 *Escherichia coli*, 33 *Klebsiella pneumoniae*, and *K. michiganensis* (KoxSC) isolates. Most samples originated from urine, followed by blood, bronchial aspirates, wound secretions, and fecal material. Nearly all isolates (94%) were recovered from patients in critical-care units, reflecting the referral-based nature of the sampling. Neonates and infants represented a substantial proportion of *Klebsiella* infections, whereas older adults were more frequently associated with *E. coli* infections (**Table 1**).

**Table 1:** Clinical characteristics and laboratory findings of resistant *Klebsiella* spp. (n=35) and *Escherichia coli* (n=30) isolates from hospitalized patients.

		*Klebsiella* spp.	*Escherichia coli*
Type of patient		N° of patients/N° of strains % (n=35)	N° of patients/N° of strains % (n=30)
	Newborn	28.5%	13.3%
	Seniors	28.5%	53.3%
	Patients with co-morbidities	11.4%	6.6%
	Patients with severe illness	97.1%	93.3%
	Co-infections with other bacteria	60%	36.6%
Sample type			
	Urine culture	51.4%	43.3%
	Bronchial aspirate	31.4%	10%
	Blood	5.7%	6.6%
	Wound discharge	11.4%	36.6%
Resistance markers			
	Potential carbapenemase-producing organisms (CPO)	28.5%	10%
	Producer of extended-spectrum β-lactamases (ESBL)	100%	100%
	MDR	48.5%	83.3%
	XDR	51.42	16.6%
	PDR	0%	0%
Antimicrobial resistance profiles			
	Amikacin	11.42%	0%
	Amoxicillin-clavulanic acid or ampicillin-sulbactam	85.7%	50%
	Piperacillin, tazobactam	65.7%	33.3%
	Cefazolin, cefoxitin, ceftriazone, cefepime or cefuroxime	97.1%	100%
	Gentamicin	57.1%	40%
	Ciprofloxacin, levofloxacin	91.4%	96.6
	Trimethoprim-sulfamethoxazole	82.8%	90%
	Imipenem, meropenem, ertapenem	31.4%	13.3%
	Tigecycline	25.7%	3.3%
	Azithromycin	80%	80%
	Chloramphenicol	48.5%	33.3%
	Tetracycline	80%	83.3%
	Aztreonam	91.4%	36.6%

†*Klebsiella* spp.: 33 *K. pneumoniae* and 2 KoxSC isolates. Neonates: age ≤30 days; Older adults: age ≥65 years. Comorbidities: diabetes mellitus, hypertension, and/or immunosuppression; individual-level data and prior antibiotic exposure were not available. CPO, carbapenemase-producing organism; ESBL, extended-spectrum β-lactamase; MDR, non-susceptibility to ≥1 agent in ≥3 classes; XDR, non-susceptibility to ≥1 agent in all but ≤2 classes; PDR, non-susceptibility to all agents tested. Susceptibility interpreted per CLSI guidelines.

Patient type distribution, sample type proportions, resistance markers (CPO, ESBL, MDR, XDR, PDR), and antibiotic resistance profiles from the BD Phoenix™ M50 panel are presented.

Co-infections were common, particularly among *Klebsiella* isolates, where 60% were recovered alongside other pathogens. *Acinetobacter baumannii, Enterococcus faecalis*, and *Pseudomonas aeruginosa* were the most frequent co-occurring organisms. Co-infection with *A. baumannii* and *Candida* spp. was restricted to XDR isolates, pointing to a respiratory critical-care niche where the most resistant strains accumulate (**Figure 1B**). In *E. coli*, co-infections were observed in 36.7% of cases, with *P. aeruginosa* and *K. pneumoniae* predominating (**Figure 1C**). The diversity of co-infecting species increased over the study period, especially in 2023, coinciding with a rise in XDR phenotypes.

**Figure 1:**
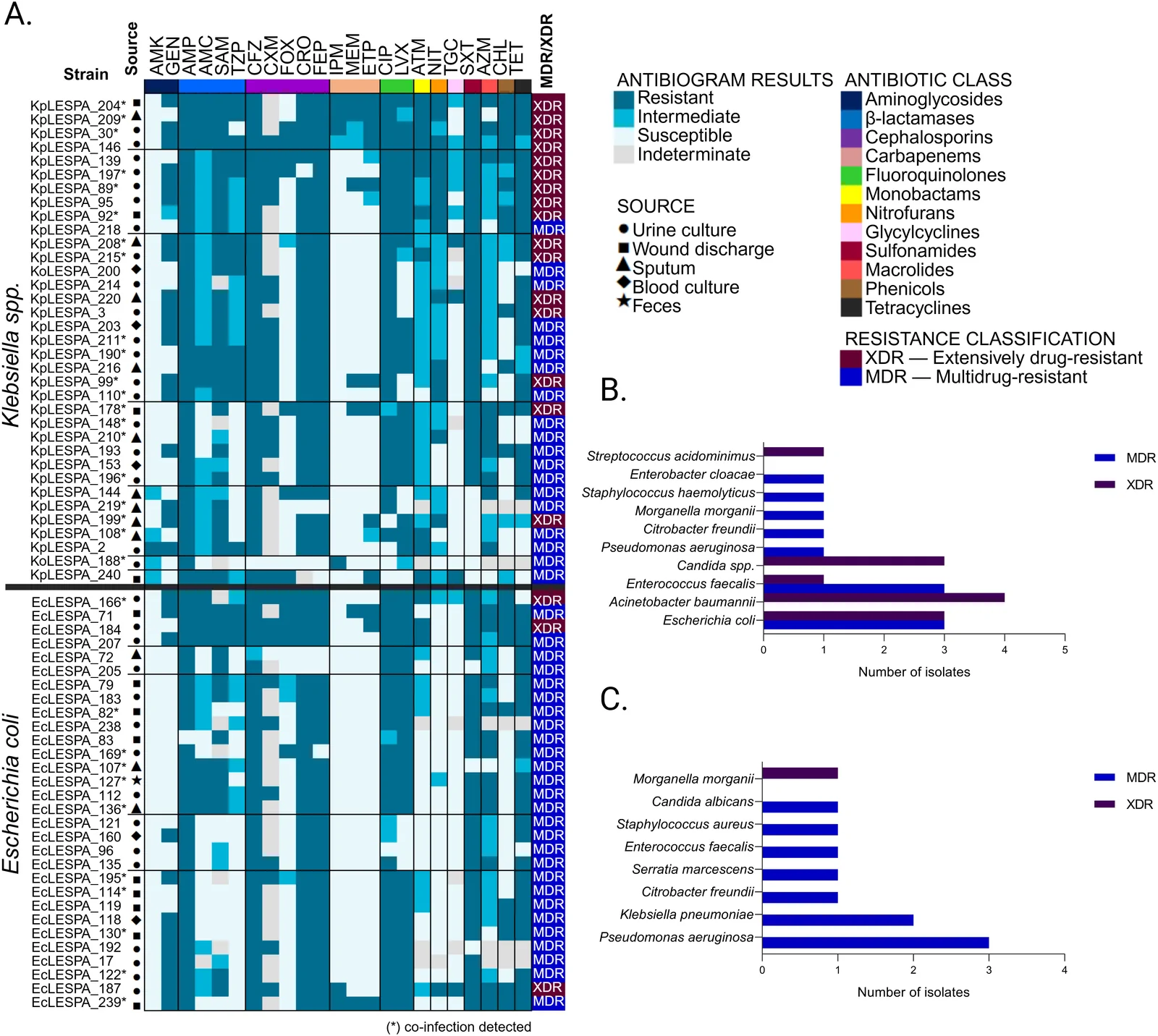
Clinical characteristics of LESPA isolates. **(A)** Antibiogram heatmap of *Klebsiella* spp. (n=35) and *E. coli* (n=30). Rows = isolates; columns = antimicrobial agents grouped by class (abbreviations defined in Methods). Isolation source: • urine; ▪ wound discharge; ▴ sputum; ♦ blood; ★ feces. (*) co-infection; MDR, multidrug-resistant; XDR, extensively drug-resistant. **(B)** Co-infecting organisms in *Klebsiella* spp. (57% co-infection rate). **(C)** Co-infecting organisms in *E. coli* (37% co-infection rate). Both panels stratified by MDR/XDR classification. Created in BioRender. Ceapa, **(C)** (2026) https://BioRender.com/b1q3cuk.

### Antimicrobial resistance profiles

Phenotypic susceptibility testing revealed a strikingly broad resistance landscape across the 65 sequenced isolates (**Figure 1A**, **Table 1**). Resistance to β-lactams—including ampicillin, amoxicillin–clavulanate, and piperacillin–tazobactam, which remain among the most frequently prescribed first-line agents in Mexico—was nearly universal in both *E. coli* and *Klebsiella* spp. Cephalosporins, particularly ceftriaxone and cefotaxime, which are widely used in emergency and inpatient care, also showed extremely high resistance rates, reaching 97.1% in *Klebsiella* spp. and 100% in *E. coli*. Fluoroquinolone resistance was similarly elevated. Ciprofloxacin and levofloxacin, commonly used in Mexico for urinary and respiratory infections, showed resistance rates above 90% in both species. Sulfonamide resistance, particularly to trimethoprim–sulfamethoxazole—a widely used outpatient therapy—also exceeded 90%, limiting the utility of this historically accessible and inexpensive drug.

Carbapenem resistance displayed a more heterogeneous pattern. While *Klebsiella* spp. showed resistance in nearly one-third of isolates (31.4%), *E. coli* exhibited lower but still concerning levels (13.3%).

Aminoglycosides, particularly amikacin, remain among the few agents with preserved activity. Amikacin resistance was limited to 11.4% of *Klebsiella* isolates and was absent in *E. coli*, consistent with its continued use as a second-line therapy in Mexican ICUs. In contrast, gentamicin—more commonly used in general wards—showed higher resistance rates, reflecting differential selective pressures across hospital units.

Tigecycline, often reserved for severe infections in Mexico due to cost and stewardship restrictions, retained activity against most isolates. However, resistance increased sharply in *Klebsiella* sputum isolates between 2022 and 2023, rising from 0% to 40%, raising concerns about emerging tolerance in respiratory settings.

Nitrofurantoin, frequently used for uncomplicated urinary tract infections in Mexico, maintained good activity against *E. coli* throughout the study period, despite its intrinsic limitations against *Klebsiella* spp. Temporal analysis revealed that resistance patterns intensified after 2020. In *E. coli*, resistance to β-lactams, sulfonamides, and fluoroquinolones remained consistently high across all years, but the emergence of XDR phenotypes was restricted to isolates collected in 2023. In *Klebsiella* spp., resistance trajectories were more dynamic, with marked increases in carbapenem and tigecycline resistance in the later years of the study.

### Genotype–phenotype concordance

The comparison between phenotypic susceptibility profiles and genomic predictions revealed substantial but imperfect agreement, consistent with the known influence of factors beyond gene presence — including gene expression levels, regulatory mutations, epistatic interactions, promoter efficiency, and experimental testing conditions — on phenotypic resistance outcomes. Overall concordance across all antibiotic classes was 78.9%, but agreement varied substantially depending on the drug class and the species involved (**Table 2**). The highest concordance was observed for fluoroquinolones, sulfonamides, and macrolides—drug classes that have historically been widely used in Mexico for outpatient and inpatient infections. In these categories, concordance exceeded 90%, reflecting the strong association between well-characterized resistance determinants (such as *qnr, sul*, and *mph* genes) and the corresponding phenotypic outcomes.

**Table 2:** Genotype–phenotype concordance of resistome analysis (SraX) for *Escherichia coli* and *Klebsiella* spp. LESPA isolates.

Drug class	Species	Concordant R (R/R)	Discordant (R/S)	Discordant (S/R)	Concordant S (S/S)	Concordance (%)
Aminoglycosides	*Klebsiella* spp	23	0	12	0	65.7
	*Escherichia coli*	12	0	18	0	40
Cephalosporins	*Klebsiella* spp	29	5	0	1	85.7
	*Escherichia coli*	30	0	0	0	100
Diaminopyrimidines	*Klebsiella* spp	23	8	0	4	77.1
	*Escherichia coli*	22	4	0	4	86.6
Fluoroquinolones	*Klebsiella* spp	34	0	1	0	97.1
	*Escherichia coli*	29	0	1	0	96.6
Sulfonamides	*Klebsiella* spp	29	1	1	4	94.2
	*Escherichia coli*	27	0	3	0	90
Carbapenems	*Klebsiella* spp	9	1	22	3	34.2
	*Escherichia coli*	0	4	0	26	74.2
Glycylcycline	*Klebsiella* spp	6	2	9	16	66.6
	*Escherichia coli*	0	1	9	16	61.5
Macrolides	*Klebsiella* spp	33	0	0	0	100
	*Escherichia coli*	25	0	2	0	92.5
Monobactams	*Klebsiella* spp	32	0	1	0	96.9
	*Escherichia coli*	6	4	3	14	74
Phenicols	*Klebsiella* spp	14	3	11	5	57.5
	*Escherichia coli*	9	1	9	8	62.9
Tetracycline	*Klebsiella* spp	29	0	3	1	90.9
	*Escherichia coli*	25	0	2	0	92.5
	Total	445	34	108	102	78.9

For each antibiotic class and species: concordant resistant (genotype R/phenotype R), concordant susceptible (genotype S/phenotype S), and discordant calls (genotype S/phenotype R; genotype R/phenotype S) are shown with per-class concordance (%). Overall concordance: 78.9% (689 total comparisons).

Concordance means Gen/Genotype. Bold character indicates the verified global average concordance rate. *Klebsiella* spp. includes 33 *K. pneumoniae* and 2 *K. oxytoca complex* (KoxSC) isolates reported jointly due to the small KoxSC sample size.

In contrast, aminoglycosides showed the lowest concordance. Several isolates carried aminoglycoside-modifying enzyme genes despite exhibiting phenotypic susceptibility. These discrepancies may reflect low gene expression, gene dosage effects, or the presence of partially functional alleles that do not confer full resistance under standard testing conditions. The divergence between genotype and phenotype in this class underscores the need for cautious interpretation of genomic predictions when aminoglycosides—particularly gentamicin and amikacin—are used as second-line therapies in Mexican hospitals.

Carbapenem concordance differed sharply between species. In *E. coli*, genomic predictions aligned with phenotypic results in most cases, consistent with the presence or absence of well-defined carbapenemase genes. In *Klebsiella* spp., however, concordance dropped markedly. Many isolates displayed phenotypic carbapenem resistance despite lacking canonical carbapenemase genes such as *blaKPC, blaNDM*, or *blaOXA-48*. Instead, these isolates carried combinations of OXA-type β-lactamases (notably *OXA-1, OXA-2*, and *OXA-11*), porin disruptions, and efflux pump systems—mechanisms increasingly recognized in Mexican hospitals as contributors to “non-classical” carbapenem resistance. This pattern highlights the limitations of relying solely on carbapenemase detection to predict resistance in *Klebsiella* spp., particularly in regions where porin-loss-mediated resistance is becoming more prevalent.

The concordance analysis also revealed that extended-spectrum β-lactamase (ESBL) genes were present in all isolates, consistent with the near-universal phenotypic resistance to third-generation cephalosporins. This alignment reinforces the dominant role of ESBLs—especially *blaCTX-M* variants—in shaping resistance patterns in Mexico, where cephalosporins remain among the most frequently prescribed antibiotics in emergency and inpatient care.

Taken together, the concordance patterns reflect both the strengths and limitations of genomic prediction in a clinical context. While genomic data reliably captured resistance to several major antibiotic classes, discrepancies in aminoglycosides and carbapenems underscore the importance of integrating genomic findings with phenotypic testing, particularly in regions like Mexico where complex resistance mechanisms are increasingly encountered.

### Clinical characteristics of public genomes

The publicly available *E. coli* and *Klebsiella* spp. genomes used for comparison displayed substantial diversity in their clinical origins, geographic distribution, and temporal span. The dataset included 139 *Escherichia coli* and 85 *Klebsiella pneumoniae* genomes isolated from human infections across Mexico, together with 44 *K. michiganensis* genomes (3 publicly available Mexican isolates and 41 global references). These genomes collectively spanned nearly four decades of sampling, ranging from isolates collected in the late 1970s to those obtained as recently as 2021.

Clinical sources were heterogeneous and reflected the broad pathogenic potential of *E. coli* and *Klebsiella* spp. in Mexican healthcare settings. Urinary isolates were particularly abundant, consistent with the high burden of urinary tract infections caused by *E. coli* and *K. pneumoniae*. However, the dataset also included genomes from fecal samples, blood cultures, respiratory aspirates, biopsies, abscesses, liver and colon tissue, conjunctival swabs, and cerebrospinal fluid. This diversity underscores the capacity of these species to cause infections across multiple anatomical sites and clinical contexts.

Geographically, the public genomes originated from at least eight Mexican states, including Mexico City, Puebla, Morelos, Sinaloa, Sonora, Nuevo León, Yucatán, and Aguascalientes. Mexico City and Puebla contributed the largest number of genomes, reflecting more established sequencing efforts in these regions (**Figure 2**). In contrast, Aguascalientes was represented exclusively by the LESPA isolates generated in this study, highlighting the novelty and importance of this dataset for filling a regional surveillance gap. Repeated sampling from locations such as Culiacán and Puebla suggests localized sequencing initiatives, but overall, the national genomic landscape remains uneven, with more than half the states having under- or no representation (**Figure 2**).

**Figure 2:**
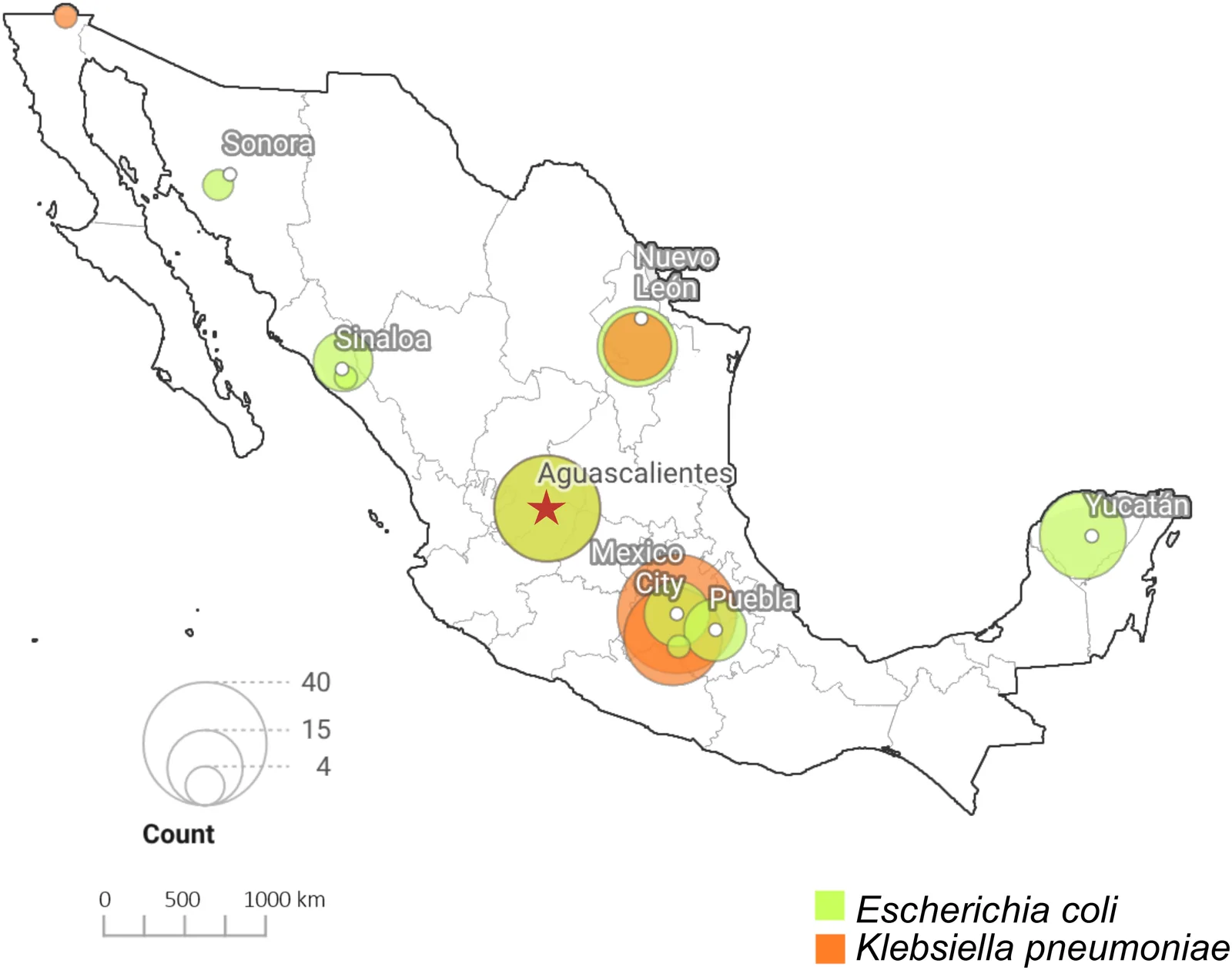
Geographic distribution of MDR *E. coli* and *Klebsiella* spp. isolates across Mexico. Bubble size reflects isolate count; colors denote species. Red star = Aguascalientes (LESPA). Created in BioRender. Ceapa, C. (2026) https://BioRender.com/ts5mw4c.

Together, the diversity and temporal breadth of the public genomes provide a robust comparative framework for situating the LESPA isolates within broader national and global patterns of *E. coli* and *Klebsiella* spp. evolution and antimicrobial resistance.

### *Escherichia coli* diversity and molecular resistance determinants

The *Escherichia coli* isolates from Aguascalientes displayed substantial genomic diversity, reflecting the circulation of multiple high-risk and regionally persistent lineages in Central Mexico. Phylogenomic analysis of 169 genomes—30 LESPA isolates and 139 publicly available Mexican genomes—showed that the LESPA strains were dispersed across the entire phylogenetic tree, with no clustering by clinical source, year of isolation, or geographic origin (**Figure 3**). Instead, isolates from Aguascalientes were intermingled with genomes from Mexico City, Nuevo León, Sonora, Yucatán, and other states, indicating that the lineages present in this region form part of broader national transmission networks.

**Figure 3:**
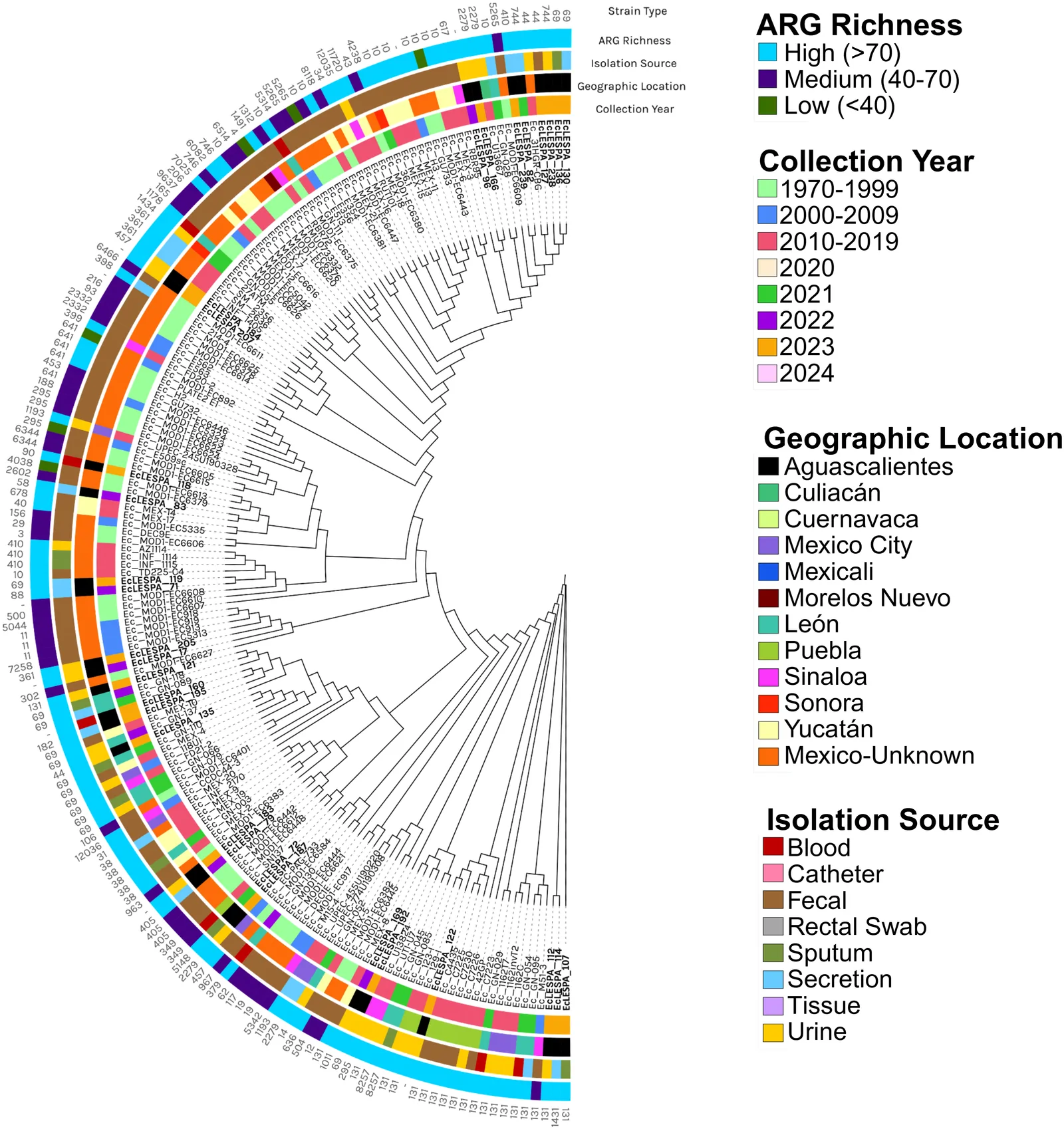
*E. coli* phylogeny (n=169). Maximum-likelihood core-genome tree; LESPA strains in bold. Outer rings: strain type, ARG richness, isolation source, location, and collection year. Created in BioRender. Ceapa, C. (2026) https://BioRender.com/r1caz7j.

The LESPA collection comprised 15 distinct sequence types (STs), including well-established high-risk lineages such as ST69 and ST131, each represented by four isolates. Notably, these same two STs were also the most prevalent among public Mexican *E. coli* genomes (ST131, n=16; ST69, n=10), confirming that the high-risk lineages circulating in Aguascalientes are not locally restricted but form part of actively circulating national transmission networks. Additional STs included ST2279, ST744, and ST44, while the remaining isolates belonged to unique or infrequently reported STs such as ST1011, ST88, ST405, ST58, ST182, ST361, ST457, ST5148, ST1431, and ST90. Six isolates could not be assigned to any known ST (sequence type) and formed distinct branches within the phylogeny. These untyped isolates carried the highest antimicrobial resistance gene (ARG) burdens in the dataset, with an average of more than 100 ARGs per genome.

Resistome clustering across all *E. coli* genomes revealed seven distinct profiles, with LESPA isolates concentrated in the clusters with the highest ARG densities, characterized by the simultaneous presence of β-lactam, macrolide, aminoglycoside, and polymyxin resistance determinants (**Figure 4**). These clusters were dominated by isolates collected after 2020, suggesting an expansion of highly resistant lineages in the post-pandemic period. Polymyxin resistance regulators — including *arnA*, *eptA*, *mgrB*, and the *pmrB/pmrC/pmrF* family — were present in nearly all LESPA isolates at frequencies substantially exceeding those in public genomes, representing a clinically significant burden given the increasing reliance on polymyxins in Mexico. The ESBL gene *blaCTX-M-15*, present in only a minority of public Mexican genomes (18%), was detected in 90% of LESPA isolates. Additional resistance genes included *blaTEM*, *blaOXA-1*, *blaOXA*-type variants, and multiple aminoglycoside-modifying enzymes.

**Figure 4:**
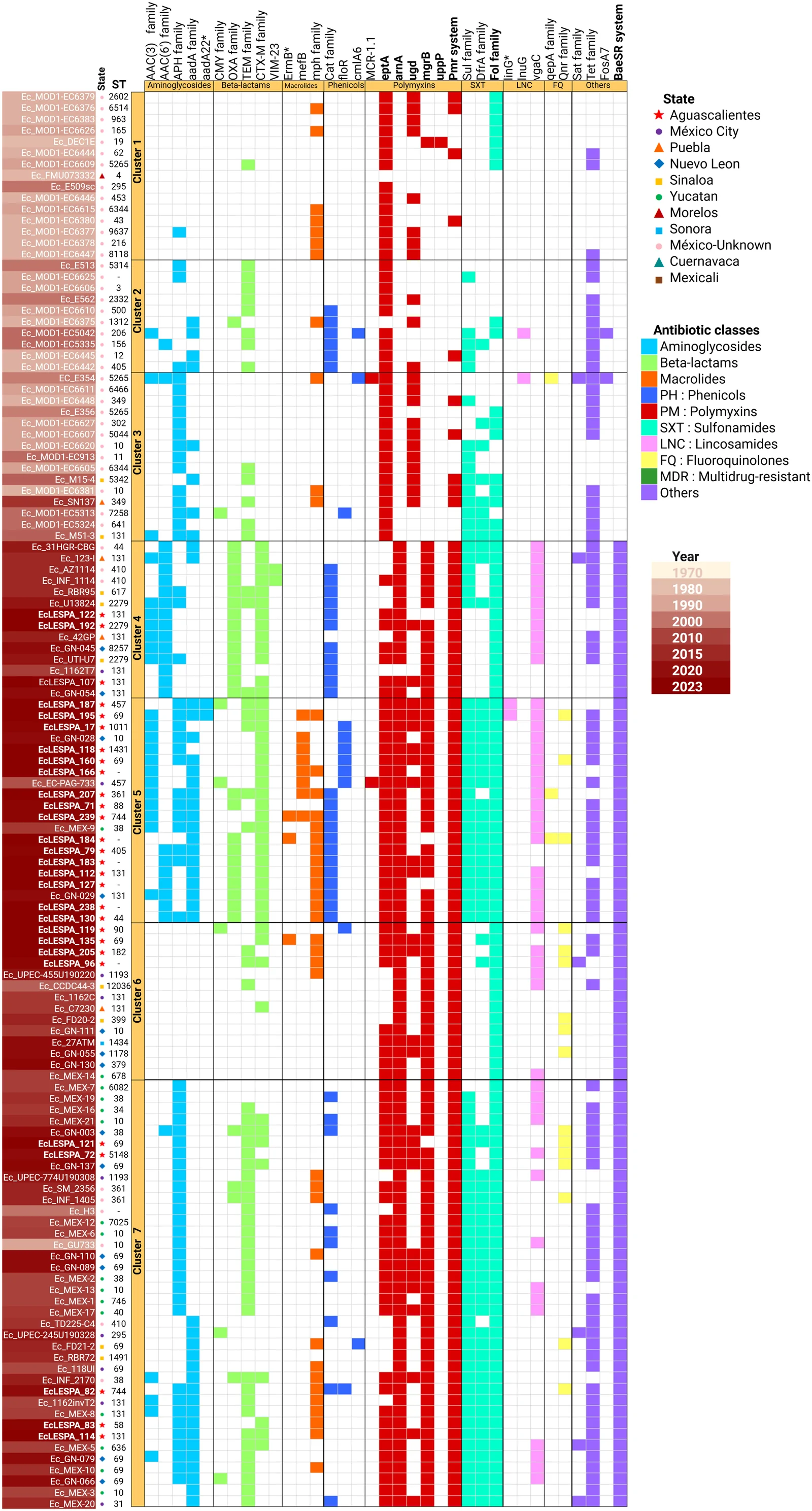
*E. coli* non-core ARG heatmap (n=169). Gene families grouped by antibiotic class. Left margin: state, ST, year. LESPA strains in bold; (*) LESPA-exclusive genes.

Mobile genetic elements were catalogued using MGE-Finder, yielding 345 elements across the 30 LESPA *E. coli* isolates, of which 26 were plasmid-associated and 319 of uncertain chromosomal origin (**Figure 5**). The single most active element was MITEEc1 (113 associations), most frequently linked to *ompT* (84 associations) and multiple virulence-associated loci, indicating a major role in shaping the outer membrane architecture of LESPA *E. coli* strains.

**Figure 5:**
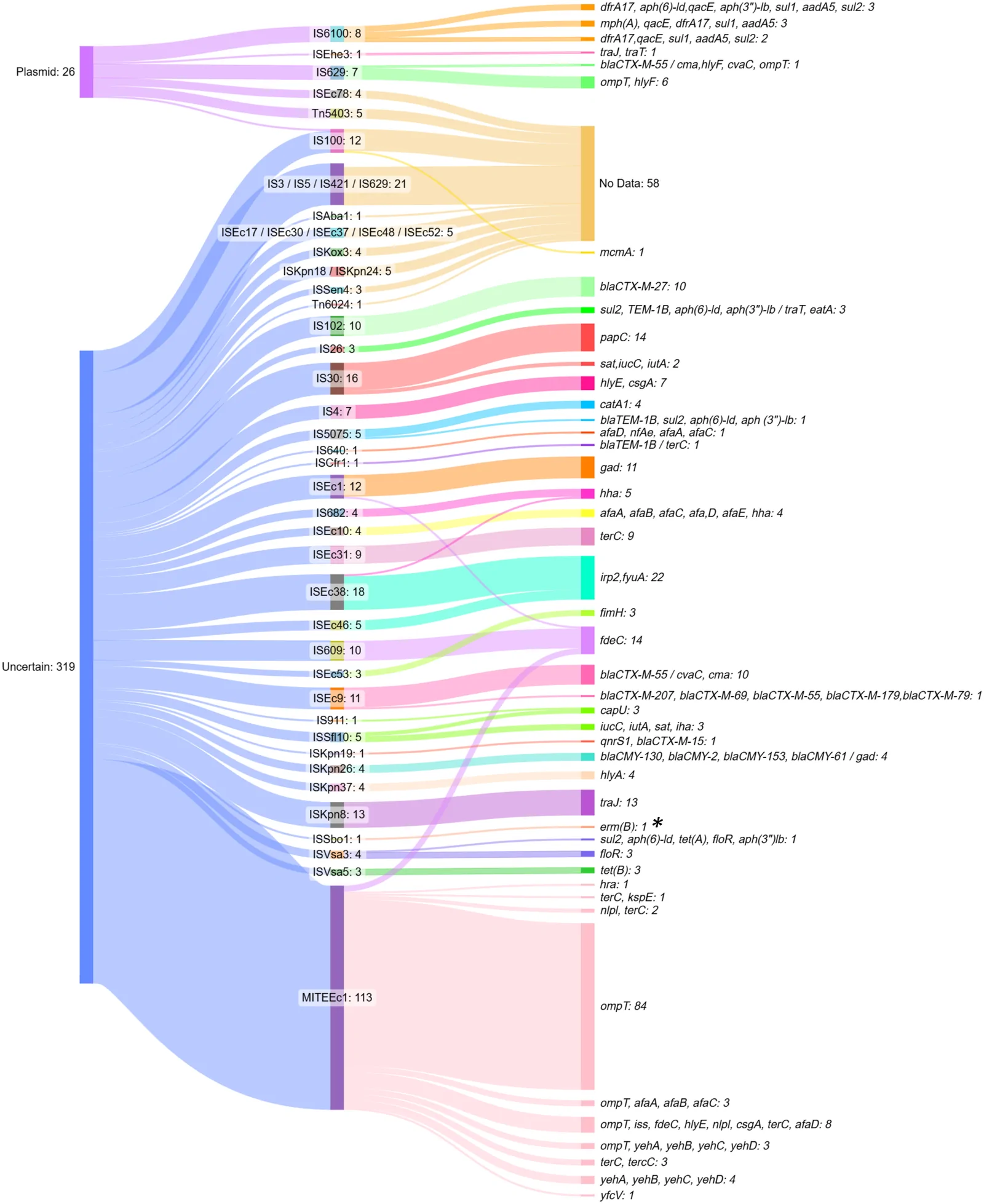
*E. coli* mobilome. Sankey diagram of MGE–gene associations in LESPA isolates (plasmid-associated n=26; uncertain origin n=319). Connection width reflects association frequency. (*) LESPA-exclusive genes.

Virulome analysis identified 253 virulence-associated units, of which 130 comprised the core virulome. These were arranged into 158 distinct profiles across 169 genomes, with LESPA genomes contributing 28 novel profiles (**Figure 6**). Seven virulence-associated units were identified exclusively in LESPA *E. coli* and absent from all previously characterized Mexican genomes: the *shu* hemin-uptake operon, *afaC-VIII*, and *galU*, indicating concurrent acquisition of resistance and virulence traits.

**Figure 6:**
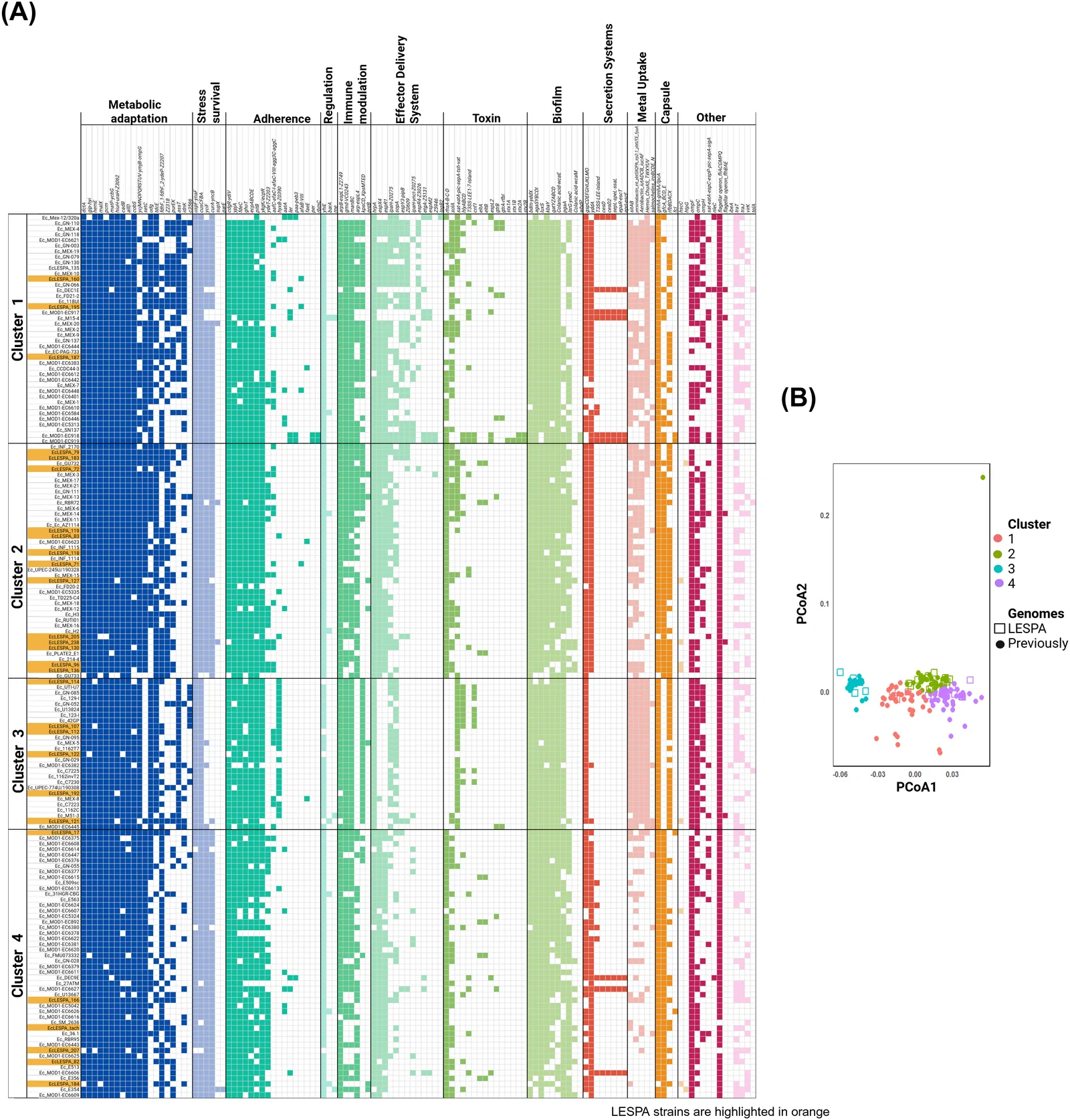
*E. coli* virulome. **(A)** Virulence gene heatmap grouped by functional category; LESPA strains in orange; four clusters delineated. **(B)** PCoA of virulome profiles; open squares = LESPA.

Together, these findings indicate that *E. coli* circulating in Aguascalientes represent a diverse and increasingly resistant subset of the broader Mexican genomic landscape. The combination of high-risk sequence types, elevated ARG richness, and widespread polymyxin-associated regulators underscores the growing complexity of *E. coli* resistance in the region and highlights the need for sustained genomic surveillance. This expansion extends to virulence.

### *Klebsiella pneumoniae* diversity and molecular resistance determinants

The *Klebsiella pneumoniae* isolates from Aguascalientes exhibited extensive genomic and phenotypic diversity, reflecting the circulation of multiple high-risk lineages within the region’s hospital network. The 33 sequenced isolates were distributed across 19 distinct sequence types, with no dominant lineage driving the local epidemiology. Instead, the phylogeny revealed a mosaic structure in which LESPA isolates were interspersed among publicly available Mexican genomes, indicating repeated introductions of diverse *K. pneumoniae* lineages rather than expansion of a single clone (**Figure 7**). This pattern mirrors the complex transmission dynamics reported in other Mexican states, where patient transfers and heterogeneous antimicrobial pressures contribute to lineage turnover.

**Figure 7:**
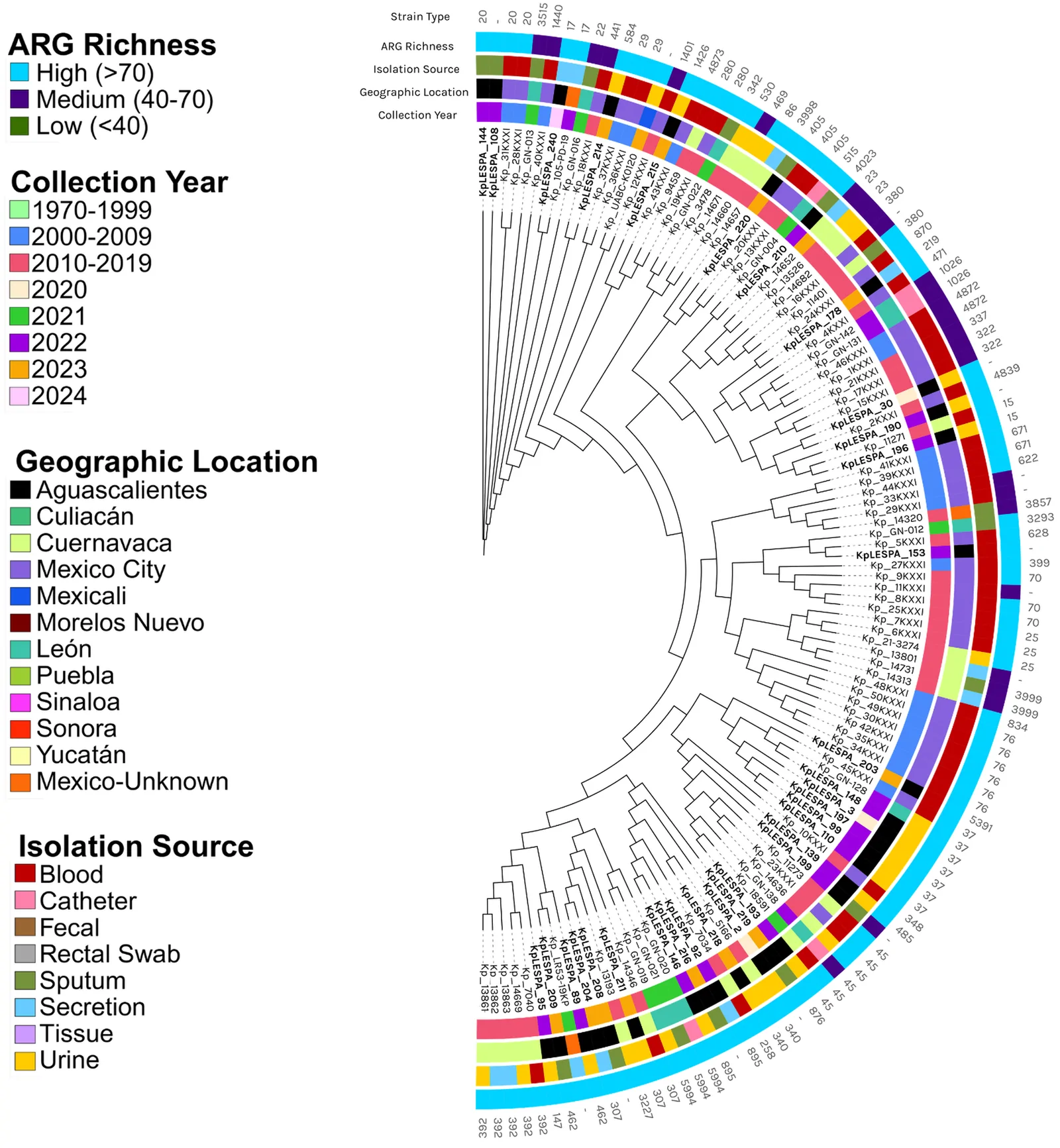
*K. pneumoniae* phylogeny (n=118). Maximum-likelihood core-genome tree; LESPA strains in bold. Outer rings: strain type, ARG richness, isolation source, location, and collection year.

Despite their phylogenetic diversity, the *K. pneumoniae* isolates shared a consistently high antimicrobial resistance burden. Many genomes carried combinations of ESBL genes—including *blaCTX-M, blaTEM*, and chromosomal *ampC*—together with OXA-type β-lactamases such as *OXA-1, OXA-2*, and *OXA-11* (**Figure 8**).

**Figure 8:**
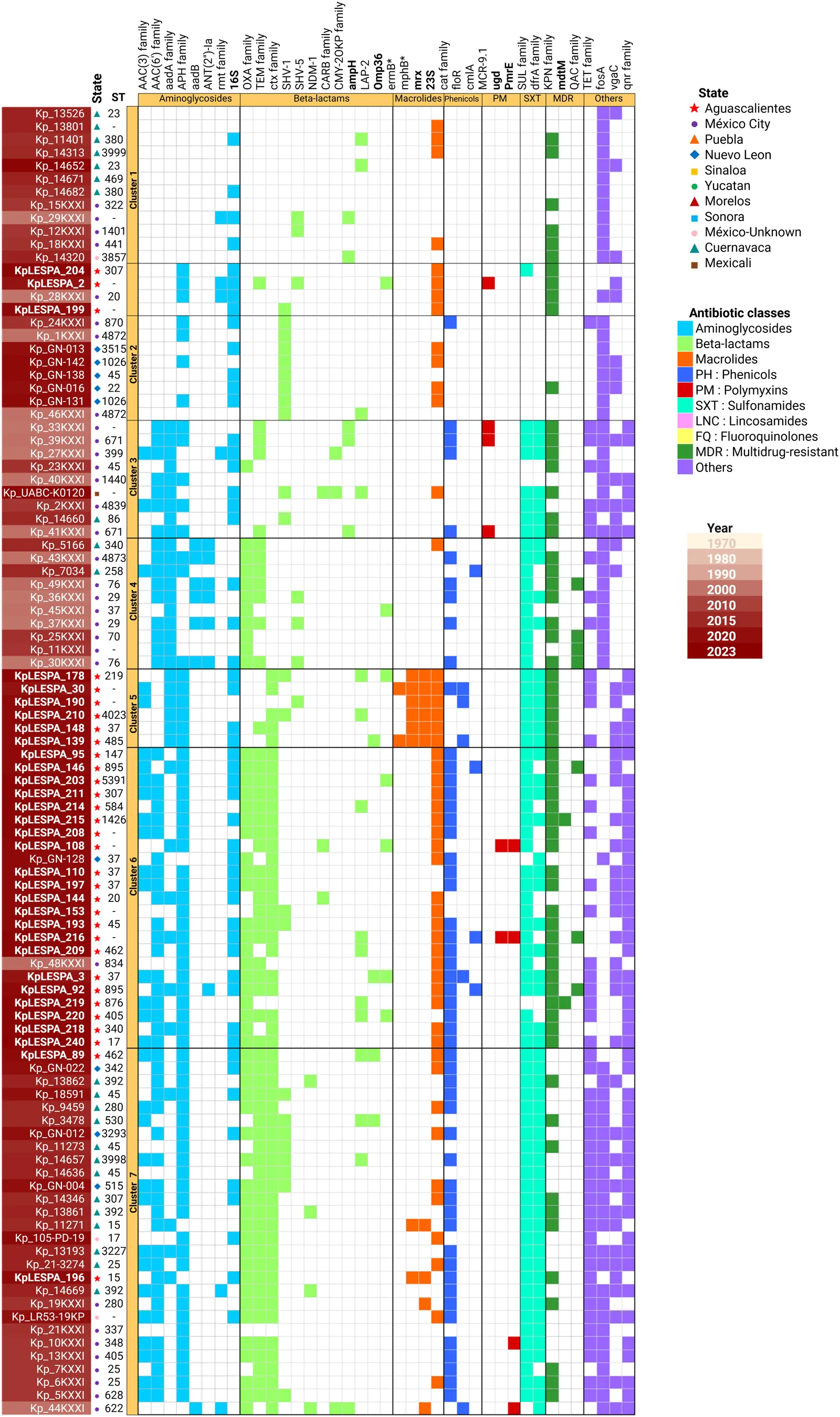
*K. pneumoniae* non-core ARG heatmap (n=118). Gene families grouped by antibiotic class. Left margin: state, ST, year. LESPA strains in bold; (*) LESPA-exclusive genes.

Mobile genetic elements were abundant across the *K. pneumoniae* genomes, with 211 MGEs identified among the LESPA isolates. Many ARGs were embedded within plasmids, transposons, and insertion sequences, suggesting active horizontal gene transfer within clinical environments (**Figure 9**). Several MGEs previously implicated in resistance dissemination were detected in novel genomic contexts, highlighting the ongoing evolution of the *K. pneumoniae* mobilome in Central Mexico. In contrast, virulome heatmap clustering grouped *K. pneumoniae* genomes into two profiles, with LESPA isolates distributed across both (**Figure 10A**), a separation confirmed by PCoA; however, this species showed no LESPA-exclusive virulence genes — a striking divergence from *E. coli*, indicating that this species diversifies primarily through resistance acquisition while maintaining a conserved virulence repertoire.

**Figure 9:**
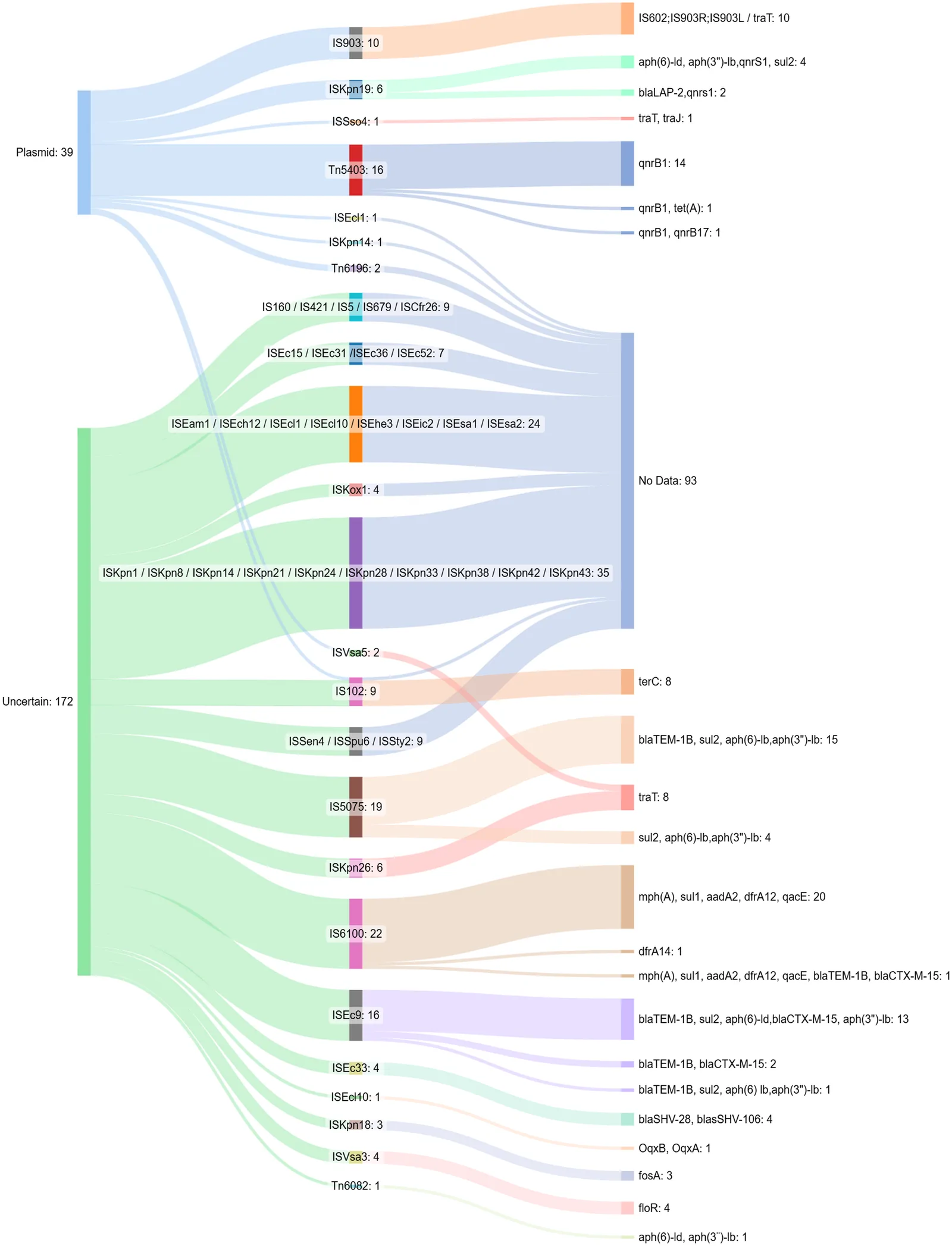
*K. pneumoniae* mobilome. Sankey diagram of MGE–ARG associations in LESPA isolates (plasmid-associated n=39; uncertain origin n=172). (*) LESPA-exclusive genes.

**Figure 10:**
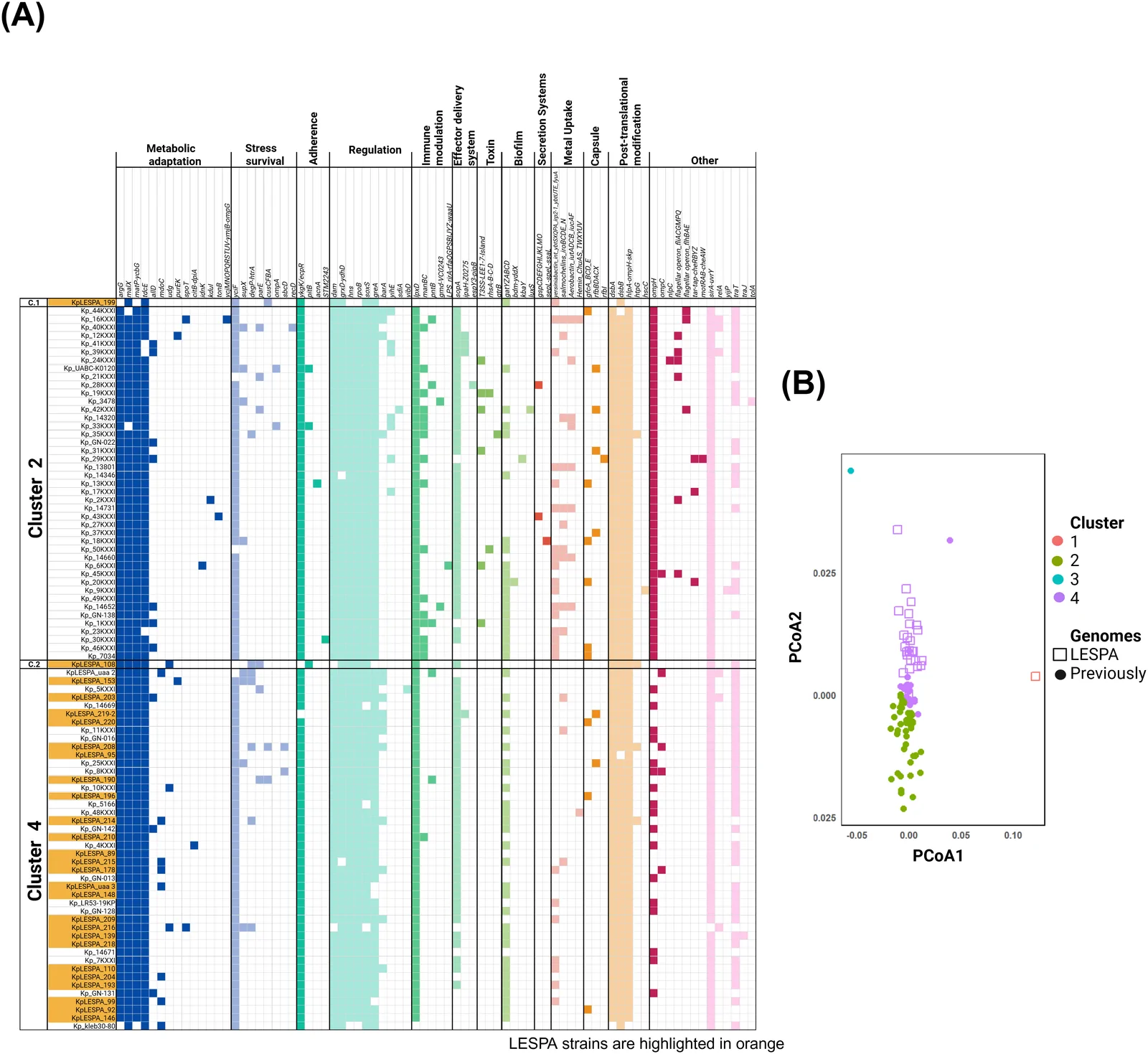
*K. pneumoniae* virulome. **(A)** Virulence gene heatmap grouped by functional category; LESPA strains in orange; four clusters delineated. **(B)** PCoA of virulome profiles; open squares = LESPA.

### *Klebsiella michiganensis* (KoxSC) diversity and molecular resistance determinants

Although there is a limited number of *K. oxytoca complex* species isolated in Mexico, they provide valuable insight into an undercharacterized pathogen. Our isolates occupied distinct branches within the global *K. oxytoca complex* phylogeny (**Figure 11A**): KoLESPA_200 (ST180) clustered with isolates from China and the USA, while KoLESPA_188 (unknown ST) grouped with Mexican and North American strains, consistent with independent introduction events. They both carry unique combinations of resistance genes, including determinants not previously documented in Mexican *E. coli* and *Klebsiella* spp. They also occupied separate resistome clusters (**Figure 11B**). Their inclusion expands the national genomic landscape and establishes a baseline for future surveillance of this MDR species.

**Figure 11:**
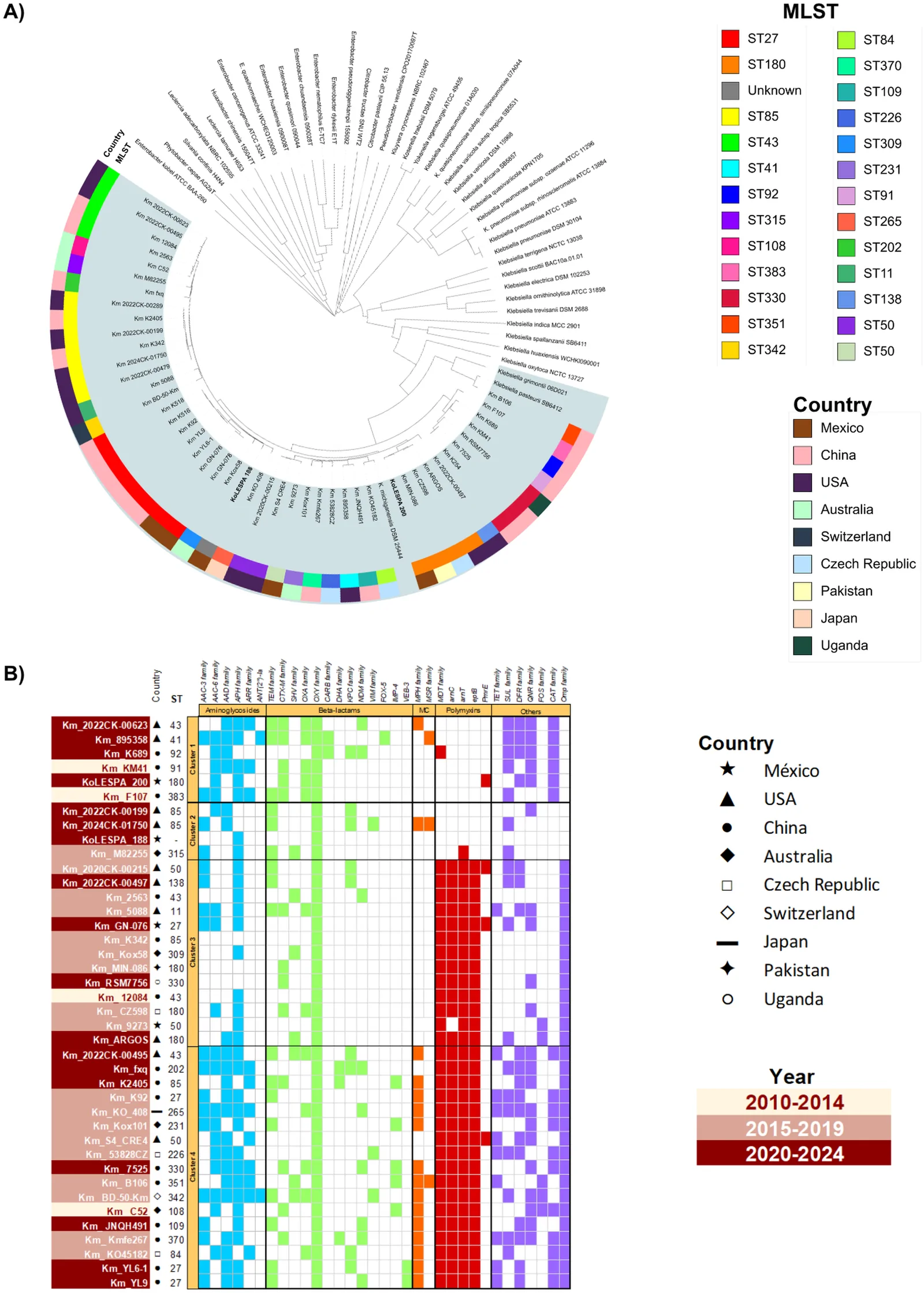
*K. oxytoca* species complex (KoxSC) phylogeny and resistome. **(A)** TYGS phylogenetic tree (n=46; 5 Mexican, 41 global); LESPA strains in bold. **(B)** Non-core ARG heatmap (n=46); left margin: country, ST, year.

These findings highlight the need for sustained genomic monitoring to track the evolution and dissemination of high-risk *Klebsiella* lineages across Mexico.

### Cross-species resistome and horizontal gene transfer overview

Pangenome analysis of the 169 *E. coli*, 118 *K. pneumoniae*, and 46 KoxSC genomes revealed open pangenomes for all three species (**Table 3**). Cloud genes — those present in fewer than 15% of genomes — represented the largest fraction in all species, accounting for 81.7% of the *E. coli* pangenome (28,042 of 34,342 genes), 76.1% of *K. pneumoniae* (18,124 of 23,801), and 63.8% of KoxSC (13,009 of 20,384), reflecting the high genomic plasticity characteristic of these clinical pathogens. Core genes shared by ≥99% of genomes numbered 1,363 in *E. coli*, 2,071 in *K. pneumoniae*, and 2,998 in KoxSC — the latter showing the highest core-to-total ratio, consistent with the more limited diversity of the KoxSC dataset.

**Table 3:** Pangenome statistics for Escherichia coli (n=169), Klebsiella pneumoniae (n=118), and K. oxytoca complex (KoxSC, n=46). Core: ≥99% of genomes; Soft core: 95–99%; Shell: 15–95%; Cloud: <15%. Roary v3.13.0, BLASTP identity threshold 95%.

Species	Total genomes (n)	Core genes (≥99%)	Soft core genes (95–99%)	Shell genes (15–95%)	Cloud genes (<15%)	Total genes
*Escherichia coli*	169	1363	940	3997	28042	34342
*Klebsiella pneumoniae*	118	2071	1079	2527	18124	23801
*K. michiganensis*	46	2998	324	4053	13009	20384

*Core genes*: present in >99% of genomes; *Soft core*: 95–99%; *Shell*: 15–95%; *Cloud*: <15%. *Analysis performed with Roary v3.13.0 (BLASTP identity threshold 95%).*

A core resistome shared across all three species was identified through comparative Venn diagram analysis (**Figure 12B**, **Supplementary Table S9**), comprising 15 ARG (antimicrobial resistance gene) families present in *E. coli*, *K. pneumoniae*, and KoxSC. These include efflux pump systems (Acr, MDT, EmrAB-TolC, AcrF, MdfA, Mar family), fluoroquinolone resistance determinants (gyrase and Par families), polymyxin resistance modulators (PhoP, PmrF), and housekeeping resistance proteins (MurA, NfsA, RpoB, CyaA, Sox family, CpxAR). Beyond this shared core, species-specific determinants were identified: OqxAB efflux pump, FosA5, and the RamAR regulatory system were shared exclusively between *K. pneumoniae* and KoxSC, while FabI, OmpK35/OmpK37, PtsI, and SHV-11 were unique to *K. pneumoniae*. In contrast, *E. coli* harbored species-specific core genes including *ampC*, *fabG*, *evgS*, *eptA*, *ompF*, and additional efflux pump components (*mdtF, mdtO, mdtP*), reflecting distinct chromosomal resistance architectures across the three species. It should be noted that detection of efflux pump gene components does not necessarily establish clinically relevant antimicrobial resistance, as functional contribution depends on expression levels and regulatory context.

**Figure 12:**
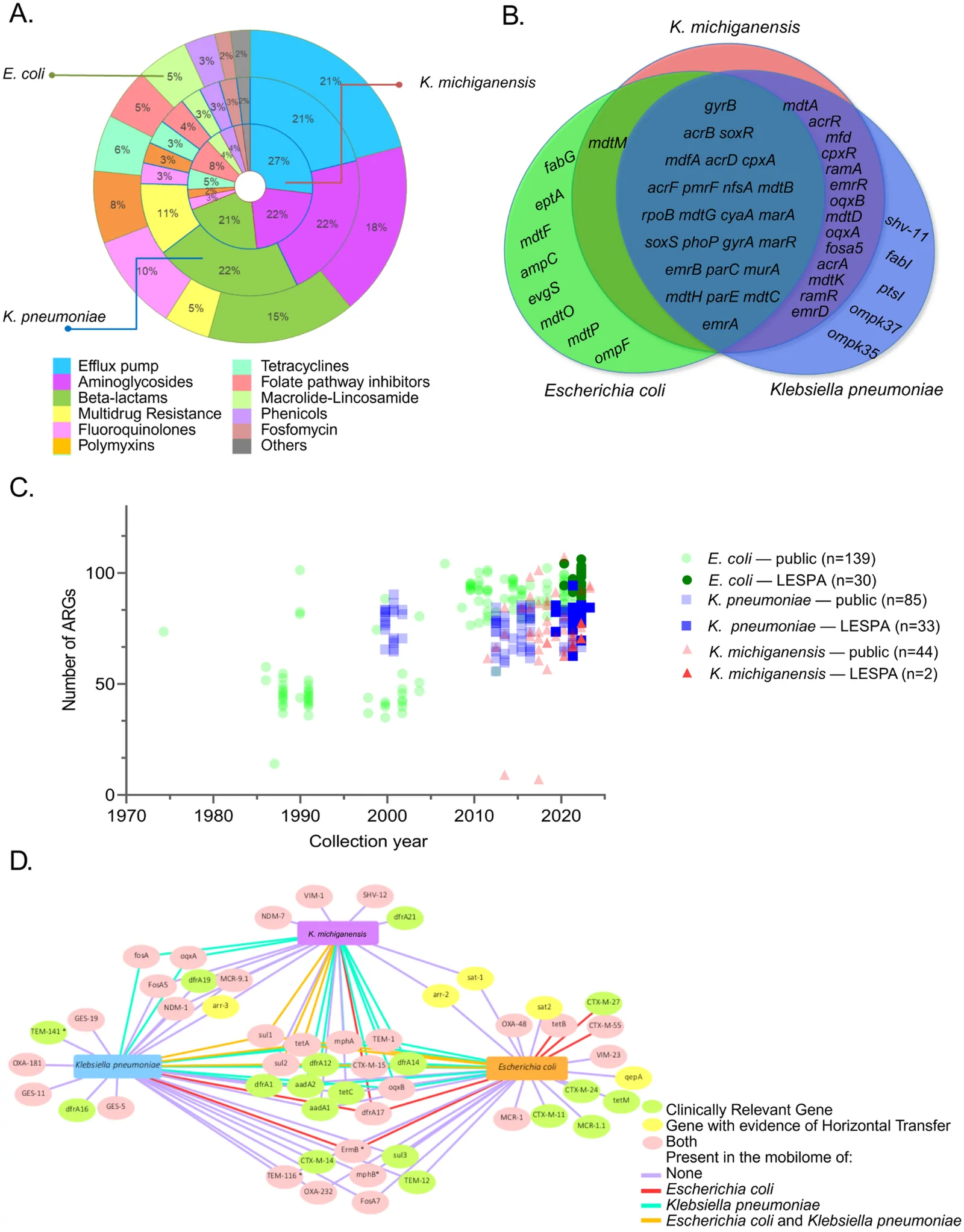
Cross-species resistome overview. **(A)** ARG functional class distribution across *E. coli* (outer), *K. pneumoniae* (middle), and KoxSC (inner rings). **(B)** Core resistance gene Venn diagram. **(C)** ARG richness versus collection year (n=333 genomes). **(D)** ARG-MGE association network of clinically relevant ARGs. Node/edge colors indicate gene category and mobilome of detection. (*) LESPA-exclusive genes.

The proportional distribution of ARG functional classes (**Figure 12A**) revealed convergent resistance architecture dominated by efflux pump systems across all three species (*E. coli* 21%, *K. pneumoniae* 22%, KoxSC 27%), followed by aminoglycosides and β-lactams. Despite this convergence, notable inter-species differences emerged: fluoroquinolone resistance genes were disproportionately represented in *E. coli* (10%) compared to *K. pneumoniae* (3%) and KoxSC (3%), while multidrug resistance genes were markedly enriched in *K. pneumoniae* (11%) relative to *E. coli* (5%) and absent in KoxSC.

Temporal plotting of ARG richness across all 333 genomes revealed a consistent escalation from 2000 to 2023, with LESPA isolates of all three species systematically occupying the upper range of the distribution (**Figure 12C**). *E. coli* LESPA strains (median 99 ARGs) substantially exceeded public Mexican genomes (median 82), with no public genome collected before 2015 reaching the ARG densities observed in LESPA 2021–2023 isolates. *K. pneumoniae* LESPA strains (median 82) similarly exceeded public genomes (median 79), with the contrast most pronounced in the 2020–2024 period. The two KoxSC LESPA isolates (79 and 72 ARGs) exceeded the international median, placing them in the upper quartile of the global KoxSC distribution.

The ARG-MGE association network (**Figure 12D**) confirmed *K. pneumoniae* as the principal ARG circulation hub, sharing genes with both *E. coli* (*CTX-M-15*, *sul1, sul2, tetA, mphA, dfrA* families — shown in orange) and harboring species-exclusive determinants (*ermB, TEM-116, TEM-141* — shown in teal). *E. coli*-exclusive mobile genes included *CTX-M-27, mphB*, and *MCR-1* variants. KoxSC uniquely carried last-resort resistance genes — N*DM-7, VIM-1*, and *MCR-9.1* — entirely absent from both *E. coli* and *K. pneumoniae* LESPA mobilomes, positioning it as a distinct reservoir of carbapenemase and colistin resistance genes.

Comparative analysis of ARG family prevalence between LESPA and public Mexican genomes revealed substantial enrichment in both species (**Figure 13**). In *E. coli* (**Figure 13A**), the most highly enriched families (Δ ≥40%) included *CTX-M-15* (90% LESPA vs. 18% public), *arnA* (100% vs. 57%), *baeSR* system (100% vs. 58%), *vgaC* (73% vs. 27%), and *pmr* family (100% vs. 60%). Eight genes were entirely absent from all public Mexican *E. coli* genomes, including *omp36* — conferring non-enzymatic carbapenem resistance — alongside *ermB*, *CTX-M-27*, *qepA*, *linG*, *aadA22*, *ramA*, and *TEM-116*. In *K. pneumoniae* (**Figure 13B**), the 23S rRNA methylase (88% vs. 32%; Δ+56%), Kpn efflux family (94% vs. 49%; Δ+45%), CTX-M-15 (85% vs. 40%; Δ+45%), and 16S rRNA methylase (79% vs. 41%; Δ+38%) were the most enriched. Twelve genes were absent from all public Mexican *K. pneumoniae* genomes, including o*mp36* of *K. aerogenes* origin (12%), *TEM-141* (3%), *ugd* (6%), and *mphB* (3%) — the largest set of nationally unreported resistance determinants identified across the three species.

**Figure 13:**
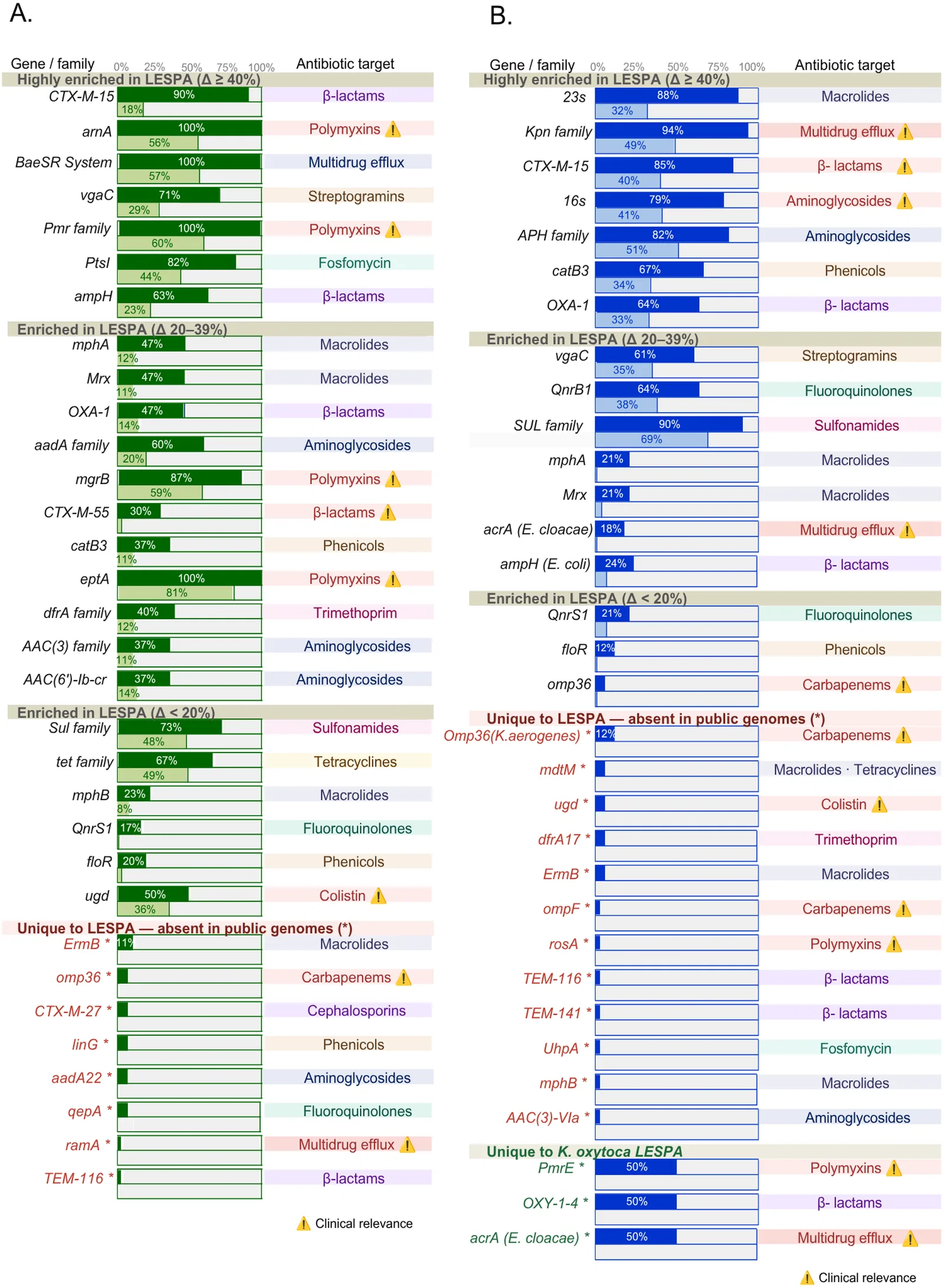
ARG family prevalence in LESPA versus public Mexican genomes. **(A)** *E. coli*. **(B)** *Klebsiella* spp. Genes stratified by enrichment level (Δ ≥40%, Δ 20–39%, Δ <20%, unique*). ⚠ last-resort antibiotic classes.

## Discussion

The genomic and phenotypic evidence generated in this study reveals an increasingly complex antimicrobial-resistance landscape among *Escherichia coli*, *Klebsiella pneumoniae*, and *K. michiganensis* (KoxSC) circulating in Central Mexico. Although the isolates analyzed represent a referral-based subset enriched for severe or treatment-refractory infections, the patterns observed provide important insight into the regional dynamics shaping AMR in clinical settings.

A notable finding is the high lineage diversity detected across the sequenced isolates, with 34 distinct sequence types and more than 100 unique resistance-gene combinations. This contrasts with the dominance of a few high-risk clones reported in large-scale surveillance efforts from Europe, Asia, and North America (Wyres et al., 2020; Murray et al., 2022; Naghavi et al., 2024), suggesting that AMR in Central Mexico is driven by multiple circulating lineages rather than clonal expansion alone. The identification of resistance determinants not previously reported in Mexican *E. coli* and *Klebsiella* spp.—including *TEM-116, aadA22, ermB*, and *uhpA*—highlights gaps in national genomic surveillance and underscores the value of expanding sequencing efforts beyond major urban centers (Acosta-Dibarrat et al., 2021; Cruz-Cruz et al., 2024).

The widespread presence of *NDM-1* and *MCR-9.1*, together with the frequent co-localization of resistance genes and mobile genetic elements, points to active horizontal gene-transfer dynamics within hospital environments. Although our analyses cannot directly infer transmission events, the recurrent association of ARGs with plasmids, transposons, and integrative elements is consistent with the rapid dissemination of resistance traits observed in other Latin American settings (Magaña-Lizárraga et al., 2019; Merida-Vieyra et al., 2019; Faccone et al., 2023; Chicoski et al., 2025). These findings reinforce the need for plasmid-focused surveillance and for integrating genomic data into infection-control workflows to detect emerging threats early.

Temporal trends further illustrate how clinical practices and structural pressures have shaped the resistome. Post-2020 isolates exhibited higher ARG richness, increased XDR rates, and more frequent coinfections, particularly in critical-care units. These patterns parallel national reports documenting intensified empirical use of broad-spectrum antibiotics during and after the COVID-19 pandemic. Carbapenemases present a substantial increase compared with pre-pandemic surveillance in Mexico, where carbapenem resistance rarely exceeded 3% (Garza-González et al., 2019). The rise was most pronounced in isolates from respiratory secretions collected in 2023, where carbapenem resistance in *Klebsiella* spp. increased from 0% in 2022 to 83% in 2023, suggesting a shift in selective pressures within critical-care environments. The sharp rise in carbapenem and tigecycline resistance among *Klebsiella* respiratory isolates in 2023 is especially concerning, as it suggests evolving selective pressures within ICU environments where antibiotic use is high and patient vulnerability is greatest.

The genotype–phenotype concordance analysis provides additional insight into the complexity of resistance mechanisms. High concordance for fluoroquinolones, sulfonamides, and macrolides reflects well-characterized genetic determinants, whereas lower concordance for aminoglycosides and carbapenems highlights the limitations of relying solely on genomic predictions for clinical decision-making. In particular, carbapenem resistance in *K. pneumoniae* may reflect an association with non-classical mechanisms such as porin loss combined with ESBL production, a pattern increasingly recognized in global surveillance studies (Morfín-Otero et al., 2013; Magaña-Lizárraga et al., 2019; Faccone et al., 2023; Chicoski et al., 2025). These discrepancies emphasize the importance of maintaining phenotypic testing capacity, especially in settings where genomic infrastructure is still developing.

Beyond the molecular findings, this study also illuminates some of the social and structural determinants that shape AMR in Mexico. Limited access to routine whole-genome sequencing, uneven distribution of microbiological capacity, and reliance on referral-based sampling create blind spots in national surveillance efforts. The high burden of coinfections—often involving *Acinetobacter baumannii*, *Pseudomonas aeruginosa*, and *Enterococcus faecalis*—reflects the challenges faced by critical-care units with constrained resources, where overcrowding, prolonged hospitalization, and invasive procedures increase the risk of acquiring MDR pathogens. These contextual factors must be considered when interpreting genomic data and designing stewardship interventions.

### Limitations

This study has several limitations that should be considered when interpreting the findings. First, the dataset reflects a referral-based sampling framework, in which isolates are submitted to the State Public Health Laboratory primarily from severe, treatment-refractory, or ICU-associated infections.

As a result, the genomic profiles described here represent a high-risk clinical subset rather than the broader population circulating in community or outpatient settings. Second, although whole-genome sequencing enabled detailed resistome and mobilome characterization, the study did not include long-read sequencing or plasmid reconstruction, limiting our ability to resolve complete mobile-element architectures and confirm transmission pathways.

Third, the number of *K. oxytoca complex* isolates was small, restricting conclusions about this species despite its clinical relevance. Fourth, genotype–phenotype discrepancies—particularly for aminoglycosides and carbapenems—highlight the need for functional validation of resistance mechanisms and reinforce the importance of maintaining robust phenotypic testing capacity. Finally, structural constraints in microbiological infrastructure across the region limit the representativeness of publicly available Mexican genomes used for comparison, underscoring the need for more equitable national genomic surveillance.

## Conclusions

The combined analyses of clinical metadata, phenotypic resistance, genomic resistome, mobilome, and virulome highlight an evolving landscape of MDR *E. coli* and *Klebsiella* spp. in Central Mexico.

High rates of coinfection with ESKAPE organisms, rising XDR trends, and pervasive ESBL prevalence among multi-drug-resistant strains pose urgent challenges for infection control. Phylogenomic interspersion of LESPA strains within national lineages demonstrates extensive interregional circulation and persistent endemic clones.

Resistome and mobilome analyses reveal that MGE-mediated horizontal gene transfer—particularly via IS26 and MITEEc1— drives ARG dissemination across species boundaries. Concurrent expansion of virulence repertoires, including novel adhesins and iron-uptake systems in LESPA isolates, may further potentiate clinical severity.

These findings advocate for integrated genomic surveillance frameworks, real-time tracking of plasmid lineages, and targeted interventions to disrupt mobile element transmission. Continued expansion of the public genome database for under-represented pathogens like *K. oxytoca complex*, coupled with standardized metadata reporting and routine virulome profiling, will be critical to forecast and mitigate future AMR and virulence threats.

## Data Availability

The datasets presented in this study can be found in online repositories. The names of the repository/repositories and accession number(s) can be found in the article/**Supplementary Material**. All genomes generated in this study have been deposited in the NCBI database under BioProject PRJNA1051187.
