# Supplementary material for: Genomic surveillance uncovers high-risk resistance profiles in Escherichia coli and Klebsiella spp. from Central Mexico
Source: Front Cell Infect Microbiol. 2026 Oct 21;16:1939440. doi: 10.3389/fcimb.2026.1939440 (PMC13730971; doi:10.3389/fcimb.2026.1939440)
Supplement: Supplementary file 1 [file SupplementaryFile1.docx]

**Supplementary Methodology for:**

**MULTI-DRUG-RESISTANT KLEBSIELLA SPP. AND E. COLI ISOLATED FROM CENTRAL MEXICO BETWEEN 2021 AND 2023, A COMPARATIVE GENETIC ANALYSIS**

**Short title: GENOMICS AND RESISTANCE OF MEXICAN DRUG-RESISTANT ENTEROBACTERIA**

**MIRIAM SARAHI LOZANO GAMBOA1*, BRIAN MUÑOZ GOMEZ1, ANDREA YAXIRI MALDONADO GONZAGA1, MOISÉS ALEJANDRO ALEJO HERNÁNDEZ1, ADRIAN CAZARES4,5, DANIEL DIAZ6, ANASTACIO PALACIOS MARMOLEJO1,3, CORINA DIANA CEAPĂ2***

1Laboratorio Estatal de Salud Pública del Estado de Aguascalientes (LESP), México.

2Microbiology Laboratory, Department of Natural Products Chemistry, Institute of Chemistry, National Autonomous University of Mexico (UNAM), Mexico.

3School of Medicine, Cuauhtemoc University, Aguascalientes Campus.

4Parasites and Microbes Programme, Wellcome Sanger Institute, Hinxton, UK.

5European Molecular Biology Laboratory European Bioinformatics Institute (EMBL-EBI), Wellcome Genome Campus, Hinxton, UK.

6Public Health Intelligence Unit, National Institute of Public Health, Cuernavaca, 62100, Morelos, Mexico

Corresponding author*: Corina Diana Ceapă, corina.ceapa@iquimica.unam.mx

**Study sites**

Clinical specimens were collected from patients at the following hospitals in the state of Aguascalientes: Tercer Milenio General Hospital, General Hospital of Zone No. 1 and No. 2 of Aguascalientes, General Hospital of Pabellón de Arteaga, General Hospital of Rincón de Romos, General Hospital of Calvillo, Women's Hospital of Aguascalientes, and Centenario Miguel Hidalgo Hospital. All specimens were forwarded to LESPA under approved biosafety and transport protocols.

**Culture Conditions**

Growth media used for each type of sample:

Urine

- Blood agar
- Orienting Chromoagar

Blood

- Blood agar
- Chocolate agar
- Orienting Chromoagar
- Candida Chromoagar

Bronchial aspirates

- Blood agar
- Chocolate agar
- Orienting Chromoagar
- MacConkey agar
- Salt and Mannitol agar
- Candida Chromoagar
- Cetrimide agar

Wound secretions

- Blood agar
- Chocolate agar
- CHROMagar Orientation
- MacConkey agar
- Salt and Mannitol agar
- Candida Chromoagar
- Cetrimide agar

**Identification by the MALDI Biotyper**

All samples received at the LESP are processed in the MALDI Biotyper® (MBT), a microbial identification system based on mass spectrometry (MALDI-TOF) that allows the impartial identification of microorganisms up to the species level. MBT determines the unique proteomic fingerprint of an organism and combines characteristic patterns with an extensive reference library to determine the identity of an organism.

**Identification using BD Phoenix™ M50 and antibiogram**

After the first identification using MALDI Biotyper® (MBT), all the samples were processed in the BD Phoenix M50 system, which uses identification and combined ID/AST panels, with 51 substrate identification wells. Antimicrobial susceptibility results were obtained using the BD PhoenixTM M50 System, which offers 85-well antimicrobial dilutions in standard AST-only panels and combined ID/AST panels. In addition, the BD Phoenix™ M50 System panels tested for key antimicrobial resistance markers, including extended-spectrum β-lactamases (ESBL), and potential carbapenemase-producing organisms (CPO). Antimicrobial susceptibility data were analyzed across 12 antibiotic classes included in our testing panel. Of these, 11 classes were considered clinically active against Enterobacterales according to CLSI/EUCAST guidelines and the Latin American consensus for defining multidrug-resistant (MDR), extensively drug-resistant (XDR), and pandrug-resistant (PDR) pathogens. [1,2]

Isolates were classified as MDR or XDR following these criteria, where MDR was defined as non-susceptibility to at least one agent in three or more antimicrobial classes, and XDR as non-susceptibility to at least one agent in all but two or fewer classes.

**Genome Selection and Re-annotation**

Publicly available genomes for all previously available clinical genomes for Mexican Enterobacteriaceae were retrieved from the BV-BRC v.3.30.19 platform (<https://www.bv-brc.org/>) (October 2024) using filters: Organism (*E. coli*, *K. pneumoniae*, *K. oxytoca*), Public: True, Genome Status: “Completed” or “WGS,” Genome Quality: “Good,” Isolation Country: Mexico, Host: Human. Selected assemblies were re-annotated with Prokka v1.14.6 [3] using the following parameters: output GFF3 format; add “gene” features for each CDS; minimum contig length 200 bp; translation table 11; e-value cutoff 1 × 10⁻⁶. Pan-genome clustering was performed with Roary v3.13.0, employing BLASTP identity threshold 95% and defining core genes as those present in ≥90% of isolates. Assembly statistics (N50, total length, GC content, and number of contigs) and genome quality metrics (completeness and contamination estimated by CheckM) were obtained directly from the BV-BRC v.3.30.19 platform (<https://www.bv-brc.org/> ) output reports (accessed March 2024). Only assemblies showing ≥90% completeness and <5% contamination were retained for downstream analyses. Per-sample coverage metrics were not calculated; assemblies were retained based on BV-BRC reported metrics as described in Supplementary Table S10.

**Phylogenomic Analysis**

Core-gene alignments generated by Roary [4] were used to infer maximum-likelihood phylogenies with IQ-TREE v2.4.0. [5] ModelFinder automatically selected the GTR + F + I + G4 substitution model based on Akaike Information Criterion. Trees were visualized and annotated in iTOL v6 [6], integrating metadata for isolation source, collection year, ARG richness, and geographic origin.

**MLST analysis**

Sequence types were assigned in silico using MLST v2.22.0 (Torsten Seemann), implemented via the Galaxy platform ([usegalaxy.org](http://usegalaxy.org)) [7]. The tool queries assemblies against the PubMLST database [8] using species-specific MLST schemes. Novel or unassigned allele combinations were reported as undetermined (–).

**Resistome Profiling**

Resistome analysis was performed using three strategies: (i) annotation in BV-BRC with CARD 4.0.0 (Dec 2024) [9], (ii) annotation in BV-BRC with NDARO (accessed Dec 2024) [10], and (iii) SraX(16) (version February 2020 and configured ARG search level set to CARD) [11]. SraX searches used DIAMOND 2.1.15. BlastX for sequence alignment, Muscle v5. for multiple-sequence alignment, with parameters: e-value ≤ 1 × 10⁻⁵, identity ≥ 85%, coverage ≥ 60%, threads = 6, ARG search level = CARD. A binary presence/absence matrix was consolidated across tools, resolving conflicts in favor of presence calls. When different tools/databases returned discordant results, we applied the following hierarchy: (i) a gene was considered present if independently detected by ≥ 2 methods (e.g., CARD + NDARO); (ii) if only one method detected the gene, it was accepted only when identity ≥ [95]% and coverage ≥ [90%]; (iii) calls failing these criteria were not included. Only the integrated results are presented into the text, the resistome profiles were analyzed using multiple approaches. Bray–Curtis distances between genomes were calculated to perform Principal Coordinates Analysis, and K-means clustering (optimal cluster number determined by elbow method) was applied. Heatmaps excluding core resistome genes (Genes in more than 90% of the samples) were generated using Microsoft Excel and other visualization tools such as Rawgraphs 2.0.1, Cytoscape 3.10.3, and Datawrapper 2.0.9.

**Genotype–phenotype concordance**

Phenotypic results from BD Phoenix™ M50 were binarized as non-susceptible (resistant and intermediate = 1) or susceptible (= 0), treating intermediate susceptibility as potential resistance in critically ill patients. ARGs were grouped by antibiotic class based on known resistance mechanisms; MDR efflux pump genes were excluded due to their broad substrate specificity. A genome was classified as genotypically resistant if ≥1 corresponding ARG was detected. Concordance per antibiotic class was calculated as the percentage of isolates with matching genotypic and phenotypic classifications. No formal statistical tests were applied; results are reported descriptively.

**Mobilome Analysis**

Mobile genetic elements were identified for each genome using MGE Finder [12], with outputs organized into pivot tables in Excel for descriptive statistics. Associations between MGEs and ARG groups were visualized via Sankey diagrams constructed in SankeyMATIC.

**Virulome analysis**

Virulence gene repertoires were annotated using BV-BRC annotations against the VICTORS [13] VFDB, and PATRIC_VF databases with thresholds of ≥90% identity and ≥80% coverage. To facilitate biological interpretation, virulence genes were grouped into virulence-associated units, including complete operons, biosynthetic gene clusters, and single virulence genes where appropriate. Only variable units — those not belonging to the core virulome — were included in the heatmaps shown in Figures 7 and 10. The resulting presence/absence matrix was analyzed by Bray–Curtis PCoA and K-means clustering (elbow method for cluster number determination). *K. oxytoca* was excluded from virulome analysis due to the limited availability of annotated reference virulence genes for this species in current databases.

**Statistical analyses**

All statistical analyses performed in this study were descriptive. Bray–Curtis dissimilarity indices were calculated to quantify compositional differences in resistome and virulome profiles between isolates. Principal Coordinates Analysis (PCoA) was applied to visualize clustering patterns. K-means clustering with the elbow method was used to determine the optimal number of resistome and virulome clusters. Concordance values are reported as percentages. No inferential statistical tests were applied.

**References:**

1. Magiorakos AP, Srinivasan A, Carey RB, Carmeli Y, Falagas ME, Giske CG, Harbarth S, Hindler JF, Kahlmeter G, Olsson-Liljequist B, Paterson DL, Rice LB, Stelling J, Struelens MJ, Vatopoulos A, Weber JT, Monnet DL. Multidrug-resistant, extensively drug-resistant and pandrug-resistant bacteria: an international expert proposal for interim standard definitions for acquired resistance. Clin Microbiol Infect. 2012 Mar;18(3):268–81. doi: 10.1111/j.1469-0691.2011.03570.x. PMID: 21793988.
2. Jiménez Pearson MA, Galas M, Corso A, Hormazábal JC, Duarte Valderrama C, Salgado Marcano N, Ramón-Pardo P, Melano RG. Consenso latinoamericano para definir, categorizar y notificar patógenos multirresistentes, con resistencia extendida o panresistentes. Rev Panam Salud Publica. 2019 Aug 22;43:e65. Spanish. doi: 10.26633/RPSP.2019.65. PMID: 31456820; PMCID: PMC6705331.
3. Seemann T. Prokka: rapid prokaryotic genome annotation. Bioinformatics. 2014 Jul 15;30(14):2068–9.
4. Page AJ, Cummins CA, Hunt M, Wong VK, Reuter S, Holden MTG, Fookes M, Falush D, Keane JA, Parkhill J. Roary: rapid large-scale prokaryote pan genome analysis. Bioinformatics. 2015 Nov 15;31(22):3691–3.
5. Minh BQ, Schmidt HA, Chernomor O, Schrempf D, Woodhams MD, von Haeseler A, Lanfear R. IQ-TREE 2: New Models and Efficient Methods for Phylogenetic Inference in the Genomic Era. Molecular Biology and Evolution. 2020 May 1;37(5):1530–4.
6. Letunic I, Bork P. Interactive Tree of Life (iTOL) v6: recent updates to the phylogenetic tree display and annotation tool. Nucleic Acids Research. 2024 Jul 5;52(W1):W78–82.
7. Seemann, T. (2014). *MLST* (v2.22.0) [Software]. GitHub.<https://github.com/tseemann/mlst>
8. Jolley, K. A., Bray, J. E., & Maiden, M. C. J. (2018). Open-access bacterial population genomics: BIGSdb software, the PubMLST.org website and their applications. *Wellcome Open Research, 3*, 124.<https://doi.org/10.12688/wellcomeopenres.14826.1>
9. Alcock BP, Raphenya AR, Lau TTY, Tsang KK, Bouchard M, Edalatmand A, Huynh W, Nguyen ALV, Cheng AA, Liu S, Min SY, Miroshnichenko A, Tran HK, Werfalli RE, Nasir JA, Oloni M, Speicher DJ, Florescu A, Singh B, Faltyn M, Hernandez-Koutoucheva A, Sharma AN, Bordeleau E, Pawlowski AC, Zubyk HL, Dooley D, Griffiths E, Maguire F, Winsor GL, Beiko RG, Brinkman FSL, Hsiao WWL, Domselaar GV, McArthur AG. CARD 2020: antibiotic resistome surveillance with the comprehensive antibiotic resistance database. Nucleic Acids Res. 2020 Jan 8;48(D1):D517–25.
10. Feldgarden M, Brover V, Gonzalez-Escalona N, Frye JG, Haendiges J, Haft DH, Hoffmann M, Pettengill JB, Prasad AB, Tillman GE, Tyson GH, Klimke W. AMRFinderPlus and the Reference Gene Catalog facilitate examination of the genomic links among antimicrobial resistance, stress response, and virulence. Sci Rep. 2021 Jun 16;11(1):12728.
11. Panunzi LG. sraX: A Novel Comprehensive Resistome Analysis Tool. Front Microbiol. 2020 Feb 5;11:52.
12. Durrant MG, Li MM, Siranosian BA, Montgomery SB, Bhatt AS. A Bioinformatic Analysis of Integrative Mobile Genetic Elements Highlights Their Role in Bacterial Adaptation. Cell Host & Microbe. 2020 Jan 8;27(1):140-153.e9.
13. Sayers, S., Li, L., Ong, E., Deng, S., Fu, G., Lin, Y., Yang, B., Zhang, S., Fa, Z., Zhao, B., Xiang, Z., Zhao, X. M., Olszewski, M. A., Chen, L., & He, Y. (2019). Victors: A web-based knowledge base of virulence factors in human and animal pathogens. *Nucleic Acids Research, 47*(D1), D693–D700.<https://doi.org/10.1093/nar/gky999>
